# Solid with infused reactive liquid (SWIRL): A novel liquid-based separation approach for effective CO_2_ capture

**DOI:** 10.1126/sciadv.abm0144

**Published:** 2022-02-09

**Authors:** Mohsen S. Yeganeh, Arben Jusufi, Shane P. Deighton, Matthew S. Ide, Michael Siskin, Aditya Jaishankar, Charles Maldarelli, Pedro Bertolini, Bharath Natarajan, Jessica L. Vreeland, Mark A. King, Andrew R. Konicek

**Affiliations:** 1 ExxonMobil Research and Engineering Company, Annandale, NJ 08801, USA.; 2 Chemical Engineering, The City College of New York, New York, NY 10031, USA.

## Abstract

Economical CO_2_ capture demands low-energy separation strategies. We use a liquid-infused surface (LIS) approach to immobilize reactive liquids, such as amines, on a textured and thermally conductive solid substrate with high surface-area to volume ratio (A/V) continuum geometry. The infused, micrometer-thick liquid retains that high A/V and directly contacts the gas phase, alleviating mass transport resistance typically encountered in mesoporous solid adsorbents. We name this LIS class “solid with infused reactive liquid” (SWIRL). SWIRL-amine requires no water dilution or costly mixing unlike the current liquid-based commercial approach. SWIRL–tetraethylenepentamine (TEPA) shows stable, high capture capacities at power plant CO_2_ concentrations near flue gas temperatures, preventing energy-intensive temperature swings needed for other approaches. Water vapor increases CO_2_ capacity of SWIRL-TEPA without compromising stability.

## Teaser

Solid with infused reactive liquid (SWIRL) is a low-energy platform for separation and reaction processes, e.g., carbon capture.

## INTRODUCTION

Anthropogenic CO_2_ emissions significantly affect climate change (*1*). Since more than 40% of the total anthropogenic CO_2_ is emitted by fossil fuel–fired power plants (*2*), an economically sound CO_2_ removal technology from power plant exhaust (flue gas) stands as one of the main challenges of our time. Although many innovative approaches to capture CO_2_ are under study such as advanced membranes (*3*), amine-functionalized mesoporous solid adsorbents (*4*), amine-appended metal-organic frameworks (MOFs) (*5*), and high surface area liquid droplets (*6*), liquid amine–based capture, introduced in 1930, is the only commercially practiced approach for capturing CO_2_ emitted by power plants (*7*, *8*). Liquid amine–based approaches offer material scalability with a well-known chemistry and high density of active sites for CO_2_ capture. However, current commercial liquid amine–based CO_2_ capture is carried out in a very large absorber tower providing a modest liquid/gas surface area–to–volume contact ratio (A/V) of about 500 m^−1^ to facilitate the gas/liquid interaction (*9*). The liquid amine is typically premixed with a high fraction of water to reduce viscosity. The high heat capacity and heat of vaporization of water add substantial energy deficits to the removal and regeneration processes (*10*). Furthermore, water dilution imposes a limitation on the ability to capture CO_2_ at the flue gas temperature (greater than 100°C) (*11*). Therefore, gas-fired power plants waste energy cooling the flue gas to below 60°C before absorption only to then reheat the spent, CO_2_-rich amine/water solution above 110°C in a regeneration tower to release the captured CO_2_ for storage and utilization (*12*). Regenerated amine is sent back to the absorber tower for repeated cycles of CO_2_ capture. Up to 80% of the energy needed in postcombustion CO_2_ capture processes using aqueous amine is spent during the solvent regeneration stage (*13*). Generally, the high-energy penalty of the operation and the costly infrastructure prohibit widespread implementation of the nearly century-old liquid amine–based technology.

Here, we report on the fabrication and demonstration of a novel liquid-based approach that is motivated by liquid-infused surface (LIS) technology. An LIS is a chemically functionalized, microtextured solid substrate that is able to trap and immobilize a liquid layer tens of micrometers thick. LISs have been observed in nature. For example, *Nepenthes* pitcher plants generate an LIS to produce a very slippery surface to capture insects (*14*). Biomimicry using LIS with nonreactive liquids has attracted much attention over the past decade for potential applications in bio- and hydrocarbon fouling prevention (*15*, *16*), corrosion inhibition (*17*), anti-icing surfaces (*18*), as well as friction and drag reduction (*19*). While applications using the infused, nonreactive liquid of an LIS to isolate or protect a solid surface have proven fruitful, exploitation of the immobilized liquid of LIS itself as a reactive medium has not yet been considered. Since the infused liquid is strongly held by capillary forces on the solid surface, it can be formed and structured by controlling and shaping the underlying substrate, enabling a new class of technological opportunities. For example, generating an LIS using a reactive liquid on a textured and chemically modified continuum solid structure with high A/V (i.e., greater than 2000 m^−1^) can shape a liquid with a similarly large A/V. We refer to this class of LIS as a “solid with infused reactive liquid” (SWIRL). The high A/V of a SWIRL could extend the applicability of LIS to low-energy, liquid-based reactions and separations, with a key example being CO_2_ capture.

We demonstrate that such a high A/V SWIRL-amine (amine being the infused, reactive, tens-of-micrometer-thick liquid) provides effective mass transport even with neat amine. The CO_2_ capture capacity of SWIRL-amine competes very well against all other materials reported in the past with remarkable stability, and its capacity further increases in the presence of water vapor without compromising stability. SWIRL-amine shows unusual physical behavior such as an increase in CO_2_ capacity with increasing temperature, providing optimum absorption temperatures close to, or above, 100°C. Similar CO_2_ capacity increases were first observed by Song *et al*. (*20*) in nanometer-thin layers and/or nanometer aggregates of amine molecules confined within the porosity of a Mobil composition of matter No. 41 (MCM-41) solid mesoporous molecular sieve and later in some other amine-functionalized (impregnated or grafted) mesoporous powder materials, with optimum absorption temperatures between 50° and 75°C (*20*–*25*). Diffusion of CO_2_ in nanometer-thin amine layers causes the unusual temperature behavior in amine-functionalized mesoporous materials (*20*–*22*, *24*, *25*). However, the temperature behavior of SWIRL-amine follows a different mechanism as we demonstrate here by microcapillary experiments and reactive molecular dynamics (MD) simulations. The enhanced CO_2_ capture at elevated temperature is a result of increased diffusion of carbamate, the CO_2_/amine product, in the micrometer-thick liquid film present in SWIRL-amine. Carbamate diffusion does not play a role in amine-decorated mesoporous solids where the amine layer is orders of magnitudes smaller. The increased carbamate diffusion in SWIRL-amine entails a highly effective mass transport enabling SWIRL-amine to use nearly all amine molecules during CO_2_ capture. This is accomplished at the temperature near that of the exhaust of gas-fired power plants without the need for mechanical mixing or water dilution. We also demonstrate that high-temperature, high-capacity absorption/desorption cycles can be achieved at full or close to isothermal conditions, obviating the need for energy-intensive temperature swings. Moreover, the high A/V of high-capacity CO_2_ SWIRL-amine and its thermally conductive support substrate would markedly decrease the size of future absorber units. SWIRL-amine–based carbon capture does therefore not require a dedicated regeneration tower and may not require heat exchangers in the absorption bed.

## RESULTS AND DISCUSSIONS

### Principle of SWIRL

**Fig. 1.**
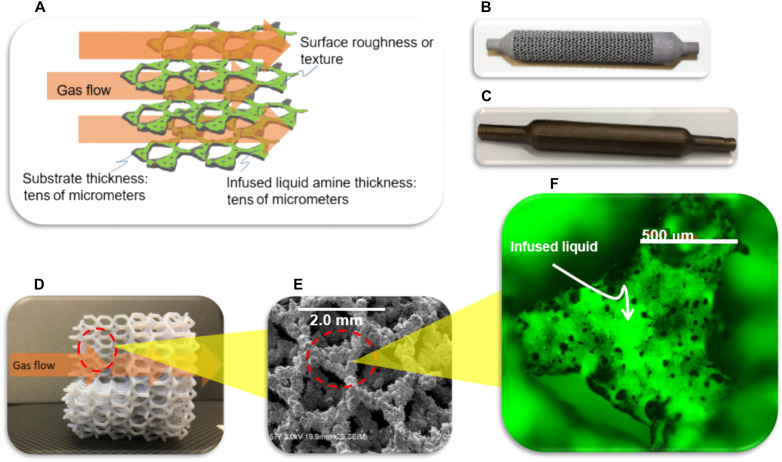
Images and schematic representation of SWIRL. (**A**) Schematic of liquid-infused amine on a textured high A/V geometry solid substrate that is in direct contact with flowing gas. (**B**) Exposed hexagonal Laves structure generated by direct metal laser sintering of AlSi_10_Mg particles (see Materials and Methods). (**C**) Same hexagonal Laves structure enclosed in a gas-tight tube that was three-dimensionally–printed around the structure for the absorption studies. (**D**) Large-scale, polymeric, printed Laves structure, for purpose of illustration and visualization [structure is approximately 60 times larger than the metallic printed SWIRL in (B) and (C)]. (**E**) Scanning electron micrograph showing the morphology of the surface structure shown in (B) and (C) (scale bar, 2.0 mm). (**F**) Micrograph of infused liquid water containing fluorescein dye on a chemically modified, exposed, hexagonal Laves structure (scale bar, 500 μm; movie S1).

The three required criteria for generating an effective SWIRL are an appropriate surface chemistry, roughness (i.e., texture), and a high A/V continuum geometry. These criteria allow the reactive liquid to spread within the texture on the solid substrate (*26*) and provide a high surface area liquid for reaction and separation applications. The surface spreading coefficient, *S*, is a measure of the propensity for the liquid to spread over the surface, which depends on the interfacial energy between the solid, liquid, and gas environment. We show that *S* > 0 is a sufficient, but not necessary, condition for the infiltration of liquid into any complex texture geometry (see the Supplementary Materials). The texture geometry determines the capillary pressure, proportional to the inverse of the characteristic surface asperity spacing, which holds the liquid within the LIS texture, and the texture depth governs the thickness, and thus the volume, of the infused liquid. A high A/V facilitates the interaction between the infused liquid and its environment through effective mass transport. We achieve the required three criteria using an additively manufactured metal (AlSi_10_Mg or 316SS) continuum structure with high thermal conductivity (*27*) where the manufacturing parameters are controlled to produce a rough surface (see Materials and Methods). The hexagonal Laves phase geometry (Fig. 1) of the substrate and surface roughness enables high A/V liquid immobilization. We obtain the wetting condition of *S* > 0 by heating the substrate in an air oven (see Materials and Methods) to remove organic contaminants and to oxidize the surface.

Nanometer-thick liquid films containing catalyst have been immobilized on powdered mesoporous materials to enhance catalytic activity for several decades. There, a few molecular layers of ionic liquid (*28*) or water (*29*) containing catalyst were spread on the surfaces of mesoporous materials for catalyst immobilization. Similarly, nanometer-thick films or aggregates of amine molecules have been confined within the porosity of mesoporous powder materials for CO_2_ capture since the early 2000s (*20*–*25*). In the above systems, gas molecules diffuse into the porosity of the mesoporous solid before reaching the active material for reaction and separation. In contrast, the infused, reactive liquid that is shaped onto the continuum solid structure of a SWIRL has a thickness more than four orders of magnitude larger than the amine layers in these past studies, hence providing a higher volume of available active material for reaction and separation. The geometry selected for the SWIRL solid substrate not only provides a high A/V but also has a high void volume fraction with small pressure drop across the bed (see the Supplementary Materials). In addition, the surface of the liquid in SWIRL is directly open to the gas phase, allowing faster interactions between the gas molecules and the liquid without the kinetically hindered diffusion caused by the mesoporous structures. SWIRL has no nanoscale channels, unlike mesoporous solid materials, eliminating the possibility for adsorption or capillary condensation of gas impurities (e.g., water), which could add to mass transport resistance and regeneration energy costs (*30*). In addition, the thermally conductive solid support of SWIRL can facilitate heat management during the exothermic absorption process. Figure 1 (A to E) presents the above SWIRL properties. Figure 1F shows a micrograph confirming the infusion of a liquid, here a water/fluorescein dye solution, into the texture of this structure. To aid the visualization, we also dispersed ~20-μm latex fluorescent beads into the water. The visible movement of the beads demonstrates the existence of a micrometers-thin layer of bulk liquid within the roughness of the solid structure (movie S1). Figure 1C shows the entire three-dimensional (3D)–printed tube that provides the gas-tight enclosure for the absorption studies.

### Capturing CO_2_ using SWIRL-monoethanolamine

**Fig. 2.**
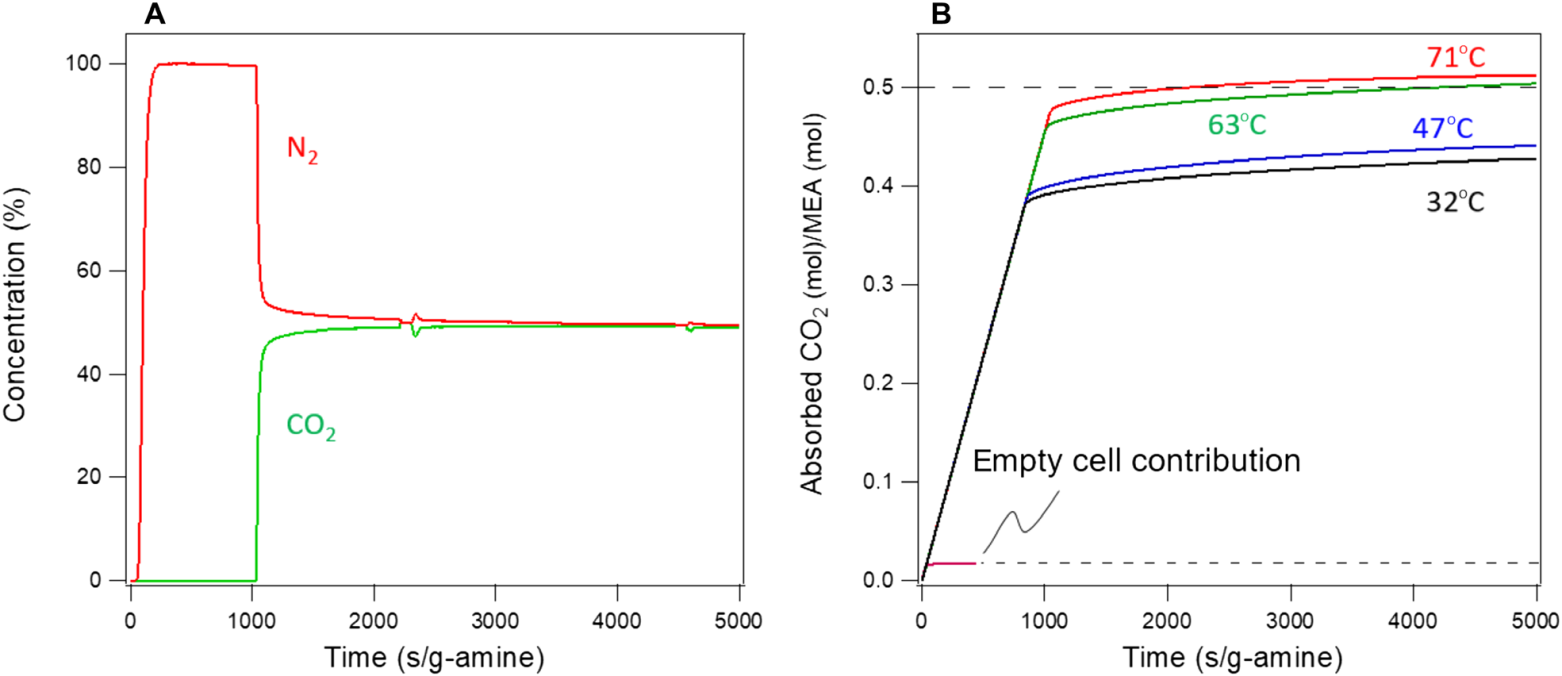
Breakthrough curve and absorption capacity of SWIRL-MEA. (**A**) Typical breakthrough curve using a SWIRL-MEA, measured at 71°C and P_CO2_ = 500 mbar. The concentration profiles are obtained by converting mass spectrometer signal intensity of each gas using a linear calibration procedure. The curves are not corrected for drift (see Materials and Methods and figs. S3 and S4). (**B**) Absorption capacity of SWIRL-MEA with normalized time at P_CO2_ = 500 mbar at different temperatures. We generate each curve by cumulatively integrating the corresponding breakthrough curves (A). The linear variations and then a sharp turn of CO_2_ capacity with time are notable in all results reported here. They are the results of the step function–like breakthrough curve (A), which is the indication of fast amine/CO_2_ kinetics in SWIRL-amine. The total capacity at each temperature also contains the empty cell contribution (i.e., no MEA) (see Materials and Methods and fig. S5). At 71°C, the CO_2_ capacity of SWIRL-MEA reaches its maximum theoretical limit for carbamate formation (excluding the empty cell contribution).

Monoethanolamine (MEA) is a well-studied molecule that contains only one primary amine. Two MEA molecules are required to react with one molecule of CO_2_ to produce one carbamate molecule in the absence of water (*24*). The simple molecular structure of MEA suggests a possibility of full utilization of this liquid amine with little steric hindrance. Thus, MEA is an excellent material to assess the effectiveness of SWIRL in accessing all amine molecules in liquid form and to understand the CO_2_ capture mechanism kinetics in SWIRL-amine. We make a SWIRL-MEA by infusing liquid MEA within the surface roughness of an oven-treated 316SS (*S* > 0) hexagonal Laves 3D-printed structure. The SWIRL-MEA is used to remove CO_2_ from a CO_2_/N_2_ (CO_2_ partial pressure P_CO2_ = 500 mbar) gas mixture under atmospheric pressure. Figure 2A shows representative concentration profiles (breakthrough curves) of N_2_ and CO_2_ emerging from the SWIRL-MEA outlet at 71°C measured with a mass spectrometer as a function of normalized time. Because of the absorption of CO_2_ in liquid amine, for a period of time, no CO_2_ molecules are detected and only N_2_ reaches the detector. Breakthrough occurs when the SWIRL is no longer absorbing all of the incoming CO_2_ and the level detected at the outlet increases. We generate similar breakthrough curves for various temperatures at P_CO2_ = 500 mbar and determine CO_2_ absorption capacity by cumulatively integrating the corresponding breakthrough curves over the time of the experiment (see Fig. 2B and figs. S3 and S4).

The above experiments present two interesting results that require further examination. First, the ratio of the moles of CO_2_ absorbed to moles of MEA in the structure (corrected for the empty cell contribution; see Materials and Methods) reaches 0.5 at 71°C and P_CO2_ = 500 mbar (Fig. 2B). The value of 0.5 is the maximum theoretical value for CO_2_ capture using MEA. SWIRL-MEA is capable of reaching this theoretical upper limit due to the accessibility of the entire, approximately 50-μm-thick, layer of liquid amine shaped into a high A/V continuum structure. The thin liquid film makes all amine molecules available for reaction with CO_2_ during a relatively short time period.

The second interesting observation is that the SWIRL-MEA CO_2_ capture capacity increases with increasing temperature. This is remarkable since the CO_2_-amine reaction is an exothermic acid-base process. Therefore, the expected behavior at higher temperature would be an increased reverse reaction rate of carbamate to CO_2_ and amine, and thus a reduction in CO_2_ capture capacity. The increase in capacity with temperature was observed only in some amine-decorated solid mesoporous powder materials in the past (*20*–*25*). The underlying mechanism of enhanced capacity with increasing temperature in amine-decorated powder mesoporous materials is an increase in flexibility of the nanometer-thick layer of amine molecules and aggregates. This enhances diffusion of CO_2_ within the amine molecules to react with the active sites (*20*–*22*, *24*, *25*, *31*). However, the SWIRL-amine liquid layer is at least 10,000 times thicker than the amine layer in an amine-decorated mesoporous powder material. This leads to a different underlying mechanism for enhanced capacity at elevated temperatures in SWIRL-amines than what was noted in the past.

We explain the observed temperature behavior in SWIRL-amine by noting the strength of the reverse reaction at elevated temperatures can be suppressed by increasing P_CO2_ (here 500 mbar). Experimental observation (presented here) suggests that the reverse reaction rate of a SWIRL-primary amine is substantially reduced at P_CO2_ as low as 100 mbar even at temperatures as high as 106°C. We also note that the CO_2_-liquid amine reaction rate is much greater than the diffusion rate of CO_2_ into the liquid amine (*32*). This leads to the nearly instantaneous acid-base reaction of CO_2_ with amine molecules near the gas/liquid interface to produce a viscous carbamate layer at the interface. The carbamate layer acts as a barrier for further interaction of CO_2_ with the remaining unreacted amine molecules in the bulk liquid. The primary mechanism of continuum CO_2_ capture by SWIRL-amines is diffusion of carbamate—not diffusion of CO_2_—into the liquid amine and diffusion of unreacted amine from the bulk to the gas/liquid interface. Higher temperatures speed up the carbamate/amine diffusion process, which makes more amine available at the surface, resulting in a higher observable CO_2_ capacity. This mechanism allows higher optimum absorption temperatures for SWIRL-amines than in previously reported materials, as we demonstrate further below. The increase in observed capacity with temperature occurs for all SWIRL-amines investigated thus far. More examples of this are presented later in the article.

To verify the above explanation and gain better insight into the kinetics, we model the interaction between CO_2_ and amine held on the SWIRL as a 1D diffusion problem. In this model, carbamate molecules are produced at a constant concentration at the gas/liquid interface and diffuse into the liquid, owing to the amine-CO_2_ instantaneous reaction at the interface. The model determines the carbamate concentration as a function of the temperature, time, diffusion rate of carbamate in the amine, and distance from the gas/liquid interface (see the Supplementary Materials). In addition, we apply a reactive MD simulation method to determine the mechanism at the molecular level (see Materials and Method and the Supplementary Materials). The reactive MD simulations indicate that CO_2_ interacts with amine molecules at the liquid/gas interface to generate carbamate. Furthermore, it shows that the penetration of CO_2_ into the bulk liquid amine is limited because of the strong interaction between amine and CO_2_, in support of the above 1D diffusion model. The simulation also demonstrates, in agreement with our 1D diffusion model, that higher temperatures increase diffusion of carbamate/amine in the system, resulting in a higher effective capacity (see the Supplementary Materials).

**Fig. 3.**
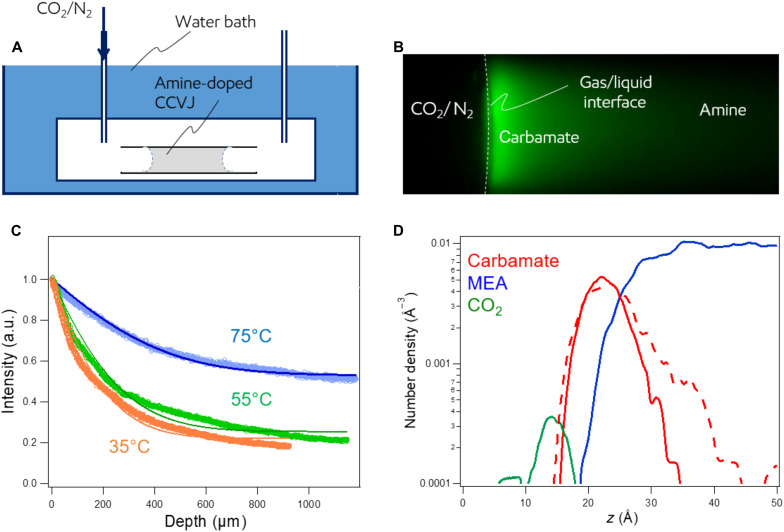
Experimental and simulation results of spatial absorption profiles of carbamate in SWIRL-MEA. (**A**) Schematic of the microcapillary/fluorescence experiment. (**B**) Fluorescence image of the microcapillary close to the gas/liquid interface after a 28-min exposure to CO_2_/N_2_ (1:1) at 35°C. The fully dark portion of the image on the left side is the CO_2_/N_2_ gas mixture; the right side of the interface is the fluorescing MEA/carbamate mixture. (**C**) Normalized intensity of the fluorescence light at the center of the capillary as a function of depth at 35°, 55°, and 75°C. Higher temperatures show improved penetration of carbamate into the amine. The solid lines through the experimental points are the fits based on the 1D diffusion model (see the Supplementary Materials). a.u., arbitrary units. (**D**) Reactive MD simulation results showing log-scale number density profiles of carbamate (red solid line), MEA (blue solid line), and CO_2_ (green solid line) at 32°C 2 ns after MEA liquid was exposed to CO_2_ gas. The results indicate that CO_2_ interacts with amine to generate carbamate at the gas/liquid interface (*z* = 15 to 20 Å). No penetration of CO_2_ in amine was observed. Increasing temperature increases diffusion of carbamate into the bulk amine (red dashed line represents the carbamate density profile at 71°C) and brings more unreacted amine molecules to the interface to react with incoming CO_2_.

We verified the 1D diffusion model and the MD simulation results by performing CO_2_ absorption experiments using a microcapillary filled with amine that was doped with 9-(2-carboxy-2-cyanovinyl)julolidine (CCVJ) (Fig. 3A; see Materials and Methods). CCVJ is a molecular rotor that fluoresces when its rotation is hindered because of a local viscosity increase. Thus, CCVJ provides a real-time visualization aid to locate carbamate formation, which has much higher viscosity than MEA. Figure 3B displays a fluorescence image of the amine-filled microcapillary after being exposed to the CO_2_/N_2_ gas mixture (see movie S2). Figure 3C depicts the normalized fluorescence light intensity profile along the center line of the capillary after a CO_2_/N_2_ exposure at various temperatures. The fluorescence light intensity profile indicates that carbamate is able to penetrate deeper into the amine at higher temperatures (see the Supplementary Materials). The solid lines in Fig. 3C are fits to the 1D diffusion model, exhibiting a good agreement of the model with the experiment. The variations of the diffusion constant of carbamate in MEA with temperature are deduced from the fitting and are tabulated in table S1. These values are in good agreement with estimated values from the Stokes-Einstein relationship (see Materials and Methods and the Supplementary Materials).

### High-temperature and high-capacity isothermal CO_2_ absorption/desorption cycle

To examine the CO_2_ removal properties at temperatures closer to the flue gas temperature and demonstrate the regeneration of CO_2_ saturated SWIRL-amine, we use a low vapor pressure liquid amine, tetraethylenepentamine (TEPA). We prepare a SWIRL-TEPA using an AlSi_10_Mg 3D-printed hexagonal Laves solid structure with the same procedure as the SWIRL-MEA described previously (see Materials and Methods).

**Fig. 4.**
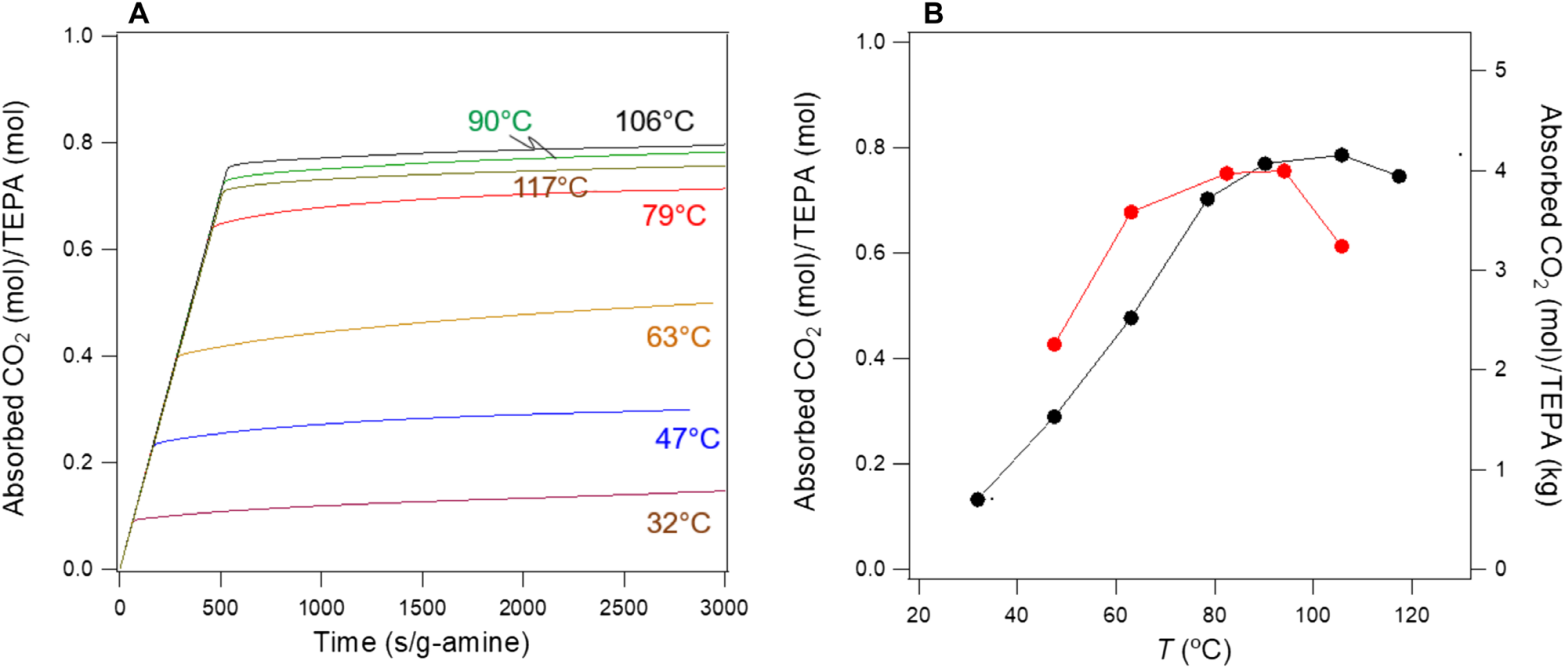
Temperature dependence of absorption capacity of SWIRL-TEPA. (**A**) Variations of SWIRL-TEPA CO_2_ absorption capacity with normalized time and temperature. We generate the absorption time profile by integrating the corresponding breakthrough data. (**B**) Temperature dependence of CO_2_ capacity of SWIRL-TEPA taken where absorption plateaus at P_CO2_ = 500 mbar (black) and 50 mbar (red). TEPA contains two primary and three secondary amines with the upper theoretical capacity of 2.5 mol CO_2_/mol TEPA. However, unlike MEA (with only one primary amine), this upper limit cannot be reached because of low activation energy of desorption of the secondary amines and high steric hindrance effects of TEPA.

Figure 4A shows the CO_2_ capacity of SWIRL-TEPA at P_CO2_ = 500 mbar as a function of normalized time for various temperatures. Each plot is obtained by integrating the corresponding breakthrough curve, which was produced after regeneration at 106°C. Figure 4B (black curve), which is generated from Fig. 4A, shows the amount of CO_2_ captured after the absorption capacity plateaus (here after 2000 s/g-amine) at various temperatures. The results prove that the CO_2_ capacity of SWIRL-TEPA, similar to SWIRL-MEA, increases with temperature. The capacity at P_CO2_ = 500 mbar peaks at approximately 106°C. The slight downward capacity at 117°C is mostly due to the competing reverse reaction of carbamate to CO_2_ and amine at this relatively high P_CO2_. Reduction in partial pressure of CO_2_, for example, to P_CO2_ = 50 mbar, further enhances the reverse reaction, and the optimum absorption temperature moves to a lower value as seen in Fig. 4B (red curve). Figure 4B (red curve) is generated from a similar absorption time profile shown in Fig. 4A, but at P_CO2_ = 50 mbar (absorption profile not shown here).

SWIRL-TEPA is capable of effectively capturing CO_2_ at low partial pressure and elevated temperatures. Its capacity of 4.0 mol/kg at 95°C/P_CO2_ = 50 mbar and 106°C/P_CO2_ = 100 mbar competes very well against previously reported solid adsorbents, which were operated at similar or even lower temperatures and/or higher CO_2_ partial pressures (see examples in Table 1).

**Table 1.** Comparison of capture capacity and multicycle stability of SWIRL-TEPA with other materials. We compare SWIRL-TEPA with TEPA-decorated mesoporous solid powders, at temperatures and partial pressures (~100°C, 50 and 100 mbar in this study) relevant to the CO_2_ conditions in power plant flue gas. In the absence of available published data at identical conditions, we used published data with the most closely matched conditions (typically with more favorable higher partial pressures and/or lower temperatures). N/R, not reported.

**Material class**	**Materials**	**P_CO2_ (mbar)**	***T* (°C)**	**mol/kg**	**Stability**	**Reference**
SWIRL-amine	SWIRL-TEPA	50	95	4.0	Yes	*
		100	106	4.0	Yes	*
Carbon molecular sieves	Activated carbon	200	100	0.24	N/R	(*50*)
Metal-organic frameworks (MOFs)	Mg_2_(dobdc)	50	95	0.8	No**	(*30*)
	Tetraamine-appended Mg_2_(dobpdc)(3-4-3)	50	90	3.4	Yes	(*38*)
Zeolites	13X	1000	70	0.36	N/R	(*51*)
	MCM-41	1000	100	0.15	N/R	(*20*)
TEPA-decorated mesoporous solid powders	TEPA-mesocellular silica foam	100	90	4.0	No***	(*22*)
	TEPA-SBA	100	75	3.30	No	(*52*)
	TEPA-mesoporous silica	50	60	3.25	No	(*21*)
	TEPA-KIL	50	90	3.0	N/R	(*53*)
	TEPA-MCM-41	120	75	3.2	No	(*54*)

*This work.

**In the presence of moisture.

***Dropped after first cycle.

The low molecular weight and high number density amine functionality of TEPA make it an attractive molecule for amine impregnation of mesoporous powder materials (Table 1). In addition, TEPA is a scalable chemical that has been used in commercial applications such as fuel additives, asphalt additives, lube dispersant, epoxy curing agents, and corrosion inhibitors. However, the weak interaction between the nanometer-thick TEPA layer and the nanoporous solid walls causes rapid cyclic leaching (*4*). For better stability, researchers have moved to higher–molecular weight amines or alternate grafting techniques, both of which reduce the optimal absorption temperature (*33*) and CO_2_ capture capacity (*4*). Alternatively, amine-appended MOFs have shown notable promise for CO_2_ capture. However, the path to commercialization of amine-appended MOFs is still evolving; these materials have poor thermal conductivity that requires substantial heat management in the adsorption bed (*34*) with uncertain material scale-up costs (*35*).

**Fig. 5.**
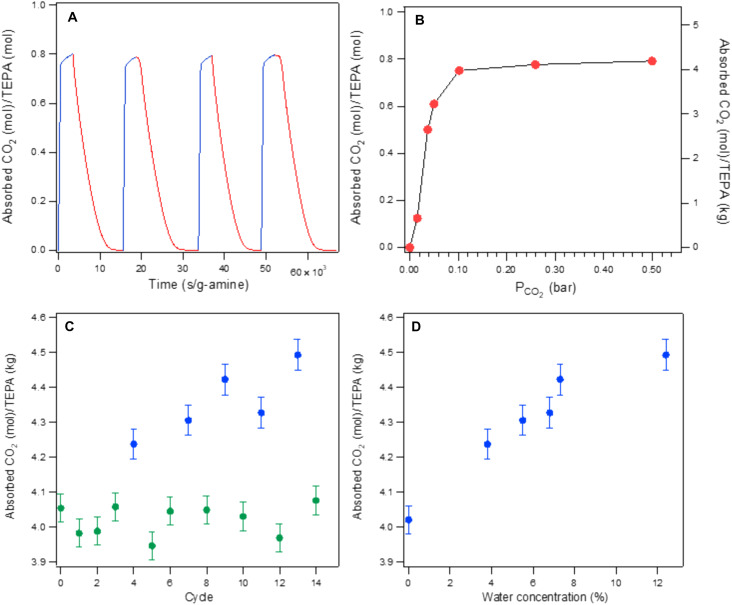
Absorption/regeneration cycles and effect of water on SWIRL-TEPA. (**A**) 106°C isothermal absorption (blue curves) and desorption (red curves) cycles of SWIRL-TEPA. The absorption curves were generated using 1:1 CO_2_/N_2_ mixture at total flow rate of 20 standard cubic centimeters per minute (SCCM). CO_2_/N_2_ flow was switched to Ar flow at 20 SCCM during regeneration. (**B**) Variations of CO_2_ absorption capacity of SWIRL-TEPA with CO_2_ partial pressure at 106°C. Little to no change of capacity is displayed for CO_2_ partial pressure greater than or equal to 0.1 bar. (**C**) Variations of CO_2_ absorption capacity of SWIRL-TEPA during a multicycle CO_2_ capture at 95°C in the presence (blue) and the absence (green) of water vapor. The N_2_:CO_2_ ratio was kept at 95:5 with a total flow rate of 50 SCCM. Water was introduced by passing the N_2_:CO_2_ gas mixture through a bubbler before entering the SWIRL. Each data point of the wet gas mixture contains a different amount of water vapor that was produced by adjusting the bubbler temperature and calculated by mass balancing of water. Variations of capacity of dry cycles after each wet cycle indicate no change in the CO_2_ capturing capability of the SWIRL. Regeneration was conducted at 105°C with Ar flow. (**D**) Variations of CO_2_ absorption capacity of SWIRL-TEPA with water concentration. Capacity improves in the presence of water vapor.

Owing to a micrometer-thick liquid layer, SWIRL-amine exhibits optimum absorption temperatures higher than those of previously reported materials. For example, the optimum absorption temperature of SWIRL-TEPA (see Fig. 4B) is 20° to 55°C higher than the absorption temperature of TEPA-functionalized mesoporous materials (*21*–*23*, *25*). The high CO_2_ capture capacity of SWIRL-TEPA at elevated temperatures offers the potential for a high-temperature, high-capacity, isothermal absorption/desorption process. Figure 5A displays an example of this. The absorption of CO_2_ occurs when a CO_2_/N_2_ mixture flows through a SWIRL-TEPA at 106°C, and a following desorption takes place when only Ar flows through the SWIRL at the same temperature. This is an example of partial pressure or concentration swing regeneration where the driver for desorption is the lack of CO_2_ in the flowing sweeping gas (*36*). In practice, other economically feasible sweeping gasses instead of Ar should be used for regeneration. Triamine-grafted pore-expanded MCM-41 (*37*) and amine-appended MOFs (*38*) are stable and show an increase in CO_2_ capture capacity in the presence of water. Sayari *et al*. (*37*) have also demonstrated other benefits of water vapor with amine, including the prevention of urea formation at elevated temperatures. For these reasons, steam- and water-containing nonadsorbing gases have been proposed as sweeping gases for the regeneration of amine-decorated mesoporous solids (*37*) and amine-appended MOFs (*38*). Figure 5C shows the CO_2_ capture capacity of SWIRL-TEPA in a cyclic absorption (95°C)/desorption (105°C) experiment with CO_2_ concentration of 5% (in a N_2_:CO_2_ gas mixture) in either the absence or presence of water vapor at various concentrations. We followed each wet cycle with a dry cycle and verified that the CO_2_ capture capacity of the dry gas mixture remains unchanged after we exposed the amine to water vapor, indicating the stability of the system. Figure 5D displays the variations of the CO_2_ capture capacity of SWIRL-TEPA with water vapor concentrations, showing improvement of the capacity in the presence of water. The increase in capacity in amine-decorated mesoporous materials (*21*, *37*) and amine-appended MOFs (*38*) can be attributed to the generation of bicarbonate, which requires fewer number of amine molecules to react with CO_2_. Figure 5C shows the remarkable stability of SWIRL-TEPA even after over 100 hours of absorption/regeneration cycles at close to 100°C with and without water vapor. We believe the stability is due to the formation of carbamate in the liquid, which substantially reduces evaporation. However, more fundamental studies are needed to better understand the underlying mechanism. The positive impact of water vapor on SWIRL-TEPA, in particular at elevated temperatures, indicates the viability of using steam or moisture-containing non-absorbing gases for the regeneration process. We have also considered that liquid loss due to the displacement of liquid amine through shear-induced drainage. The amount of liquid removed from a textured surface is directly related to the value of the shear at the surface (*39*). However, the low viscosity and velocity (~1 m/s) of flue gas generate a weak shear (~0.1 Pa), leaving the vast majority of the liquid within the surface roughness of SWIRL.

To demonstrate a broader applicability of SWIRL-amine, we measured an absorption isotherm for a SWIRL-TEPA at 106°C for CO_2_ partial pressures ranging from 500 mbar down to 15 mbar (Fig. 5B). There is little change in CO_2_ capacity from 500 to 100 mbar, indicating that the reverse reaction rate of the primary amine is negligible at 106°C for P_CO2_ greater than 100 mbar. SWIRL-TEPA removes 2.6 mol CO_2_/kg amine at 106°C even at P_CO2_ = 37 mbar. Higher capacity at lower partial pressures can be restored by slightly reducing the operating temperature. This is demonstrated for SWIRL-TEPA, where we obtained a higher CO_2_ capture capacity at P_CO2_ = 50 mbar by lowering the temperature from 106° to 95°C (Fig. 4B). P_CO2_ = 50 mbar represents the CO_2_ partial pressure of a natural gas–fired power plant exhaust.

Since CO_2_ capture capacity is such a strong function of temperature, managing the heat generated by the exothermic absorption process is critical in scaled-up applications. As the thermal conductivity of all the amine-functionalized mesoporous solids and amine-appended MOFs is intrinsically low, thermal management requires the use of an embedded heat exchanger in their adsorption beds. The size of the heat exchanger strongly depends on the difference between the operation temperature and the cooling source. It was noted that operating at ~100°C, akin to the operation temperature of SWIRL-TEPA, reduces the reactor bed size by close to fivefold, as compared to a solid sorbent adsorption bed (*5*). In addition, having a thermally conductive solid within an absorption bed, similar to SWIRL-amine, could increase in-bed heat transfer and potentially remove any need for a heat exchanger (*40*). Corrosion is not an important factor in SWIRL-amine at the noted temperatures because corrosion in amine systems is aqueous in nature (*41*), caused by free ions generated by amine or amine-degradation by-products in water (*12*). SWIRL solid support materials (including stainless steel used in this study) and the negligible presence of water in bulk liquid amine, in particular at these elevated temperatures, make corrosion less of a concern.

Our results indicate that SWIRL could provide a novel and alternative low-energy platform for critical liquid-based separation and reaction processes, such as carbon capture. Our proposed SWIRL-amine technology does not need a dedicated regeneration tower. In a scaled-up operation, SWIRL-amine would be used in a multibed system. For example, in a two-bed system, each bed alternatively operates in absorption or regeneration mode to ensure a continuous operation of the CO_2_ capture process. Overall, the potential for removing the need for heat exchangers, for any pumps to flow liquid between towers, for trays, or packing contactors (normally used in commercially practiced liquid amine towers), combined with the high A/V and high optimum operation temperature of SWIRL, offers a substantial reduction of the envisioned SWIRL bed size and the corresponding capital costs. Furthermore, high-temperature, high-capacity, isothermal cycles without the need of water dilution of amine create an opportunity to enhance the energy efficiency, and thus reduce the operational cost, of liquid amine–based carbon capture. The process can be further optimized based on the specific CO_2_ point source by modifying parameters such as the operational temperature.

## MATERIALS AND METHODS

### Additive manufacturing materials and process

The 3D-manufactured structures were created by direct metal laser sintering (DMLS) using an SLM125 Selective Laser Melting metal 3D printer. The DMLS parameters, in particular the laser energy and exposure time, are selected to ensure an adequate final surface roughness. The additively manufactured structured supports were designed using a combination of SolidWorks, nTopology, and Autodesk Netfabb Ultimate 3D computer-aided design software. The used metal powder particle sizes range from 15 to 45 μm for the AlSi_10_Mg or 316SS material.

In this work, a bioinspired hexagonal prism was chosen as an example of a lattice that meets the required criteria of high A/V and mechanical strength (see the Supplementary Text). The structure unit cell was generated within the bounds of a solid object, and any lattice edge overhanging the solid object was trimmed. This design mimics the AB_2_ structures commonly found with a hexagonally arranged Laves phase. One of the most common examples of a hexagonal Laves structure is MgNi_2_ (*42*). For this work, a 1.4-mm unit cell was generated with a 250-μm strut thickness. Figure S2 (A to C) shows views from various angles of the hexagonal Laves phase 1.4-mm unit cell.

The addition of the outer shell greatly reduces potential bypass because of the wrapping and loading of an open lattice structure. The enclosed object shown in fig. S2D was designed to facilitate the connection with the experimental setup and retain the fidelity of the lattice inside. Figure S2E shows an inside view of the symmetrical final part design before printing. The objects were unioned together using a Boolean operation, and the surface triangulated mesh was then sliced with a layer size of 30 μm using Materialise Magics before printing. The enclosed final object is 140 mm in height with a diameter of 12.7 mm and ends sized to 6.3 mm with a resulting structure A/V calculated at 2300 m^−1^.

### Preparation of a SWIRL-amine

To increase the surface energy of the solid structure, additively manufactured SWIRL (see Fig. 1) is heated in an air oven at 400°C for 3 hours while flowing air through the tube at approximately 20 ml/min. The increased surface energy ensures a positive surface spreading coefficient (see the Supplementary Text), causing the liquid to readily wet the surface and by infusing within the roughness. The structure is allowed to cool to room temperature, and then the amine is pipetted through the structure, recycling the liquid through several times. Nitrogen is then blown through the structure to remove excess amine, until no more amine exits the tube.

### CO_2_ capture experimental setup and data analysis

The inlet of the SWIRL-amine tube is connected to a 1.59 mm stainless steel gas line flowing 20 standard cubic centimeters per minute (SCCM) argon (gas flows are regulated by Brooks 5850E mass flow controllers), a thermocouple is secured to the wall of the tube, and the unit is wrapped in aluminum foil and placed inside an aluminum heating block. The outlet of the structure is connected to a Pfeiffer Vacuum Omnistar (model GSD 301) mass spectrometer. Argon is allowed to flow through the sample for approximately 30 min to purge the atmosphere, and then the sample is heated to the desired temperature. After the temperature is stabilized, the Ar flow is switched to a mixture of CO_2_/N_2_ (the ratio of CO_2_ and N_2_ flow rate is set for a desired P_CO2_) with total flow rate of 20 SCCM at a preset temperature. The mass spectrometer signal intensities for water, nitrogen, oxygen, argon, and carbon dioxide are collected for the duration of each breakthrough experiment. To remove the effect of the variation of amine mass on the concentration curves, in each SWIRL-amine preparation, we normalize the time to the mass of the amine. Figure S3 shows a flow schematic for the CO_2_ absorption experimental setup. Because of the relatively high vapor pressure of MEA, we did not attempt to demonstrate the regeneration of the saturated SWIRL-MEA at high temperatures. Instead, we used a lower–vapor pressure liquid amine, TEPA (boiling point of 340°C). We observed that an initial cycling period of absorption and regeneration of SWIRL-TEPA is necessary to stabilize the measured amine capacity. This, we believe, is due to the evaporation of volatile impurities in the amine. To this end, we recorded the breakthrough curves of SWIRL-TEPA after several cycles of absorbing at 32°C and regenerating at 106°C. After the purification cycles, the reproducibility of the breakthrough curve is smaller than 2%.

The mass spectrometer CO_2_ signal intensity tends to drift with time (see fig. S4). To correct for the drift, we switch to the bypass several times after CO_2_ has broken through. Using the bypass data, we calculate an exponential fit that we use to correct for the CO_2_ signal intensity drift.

To obtain the amount of CO_2_ absorbed (in mole) into the SWIRL, we first normalize the breakthrough curve to the intensity of the input CO_2_ gas (obtained from bypass measurements). A normalized output of zero means full CO_2_ capture, and an output value of one indicates zero capture and all of the input CO_2_ leaves the SWIRL. The normalized CO_2_ as a function of time per gram of amine for SWIRL-MEA at 32° and 71°C are shown in fig. S4B. The cumulative integral of the normalized breakthrough curve is then computed from *t*_0_ (determined when Ar is replaced by N_2_ signal breaking through the cell and being detected by the mass spectrometer) to *t*_f_ (time at the end of the experiment). We then convert the cumulative integral from total flow in cubic centimeters to moles of the CO_2_ absorbed divided by moles of amine. The conversion factor is 10^−6^*M*_w_ *q P*/60*RT* (where *M_w_* is the amine molecular weight in g·mol^−1^, *q* is the CO_2_ flow rate in cc·min^−1^, *P* is the pressure in Pa, *R* is the gas constant in J·K^−1^·mol^−1^, and *T* is the temperature in K). It is calculated from the ideal gas law based on the known flow rate, sensor pressure, and temperature. Figure S4C shows the cumulative integrals, indicating absorbed CO_2_ (mole)/amine (mole), for SWIRL-MEA at 32° and 71°C experimental runs.

### Empty cell contribution

The effect from the empty cell to the CO_2_/N_2_ flow is measured to establish the cell contribution to the breakthrough curves. The cell is cleaned as before and, under an N_2_ flow of 10 SCCM, the CO_2_ flow at 10 SCCM into the cell is established. *t*_0_ is determined when a downward variation in the mass spectrometer signal of N_2_ is observed. In the presence of amine, the Ar signal is negligible in the dynamic absorption region of the breakthrough curve. Therefore, a two-gas normalization (N_2_ and CO_2_) can be used. However, in the absence of amine, the dynamic part of the breakthrough curve occurs at a very early stage. Using Ar adds the presence of a third gas in the system and necessitates a much more complex calibration procedure. To simplify the system, we do not use Ar as sweeping gas in the absence of amine. The breakthrough curve of an empty cell is measured at various temperatures from 32° to 106°C (see fig. S5). No noticeable difference is observed between these curves, indicating that the entire breakthrough curve of an empty cell is solely due to the gas flow into the cell.

### Microcapillary/fluorescence experimental procedure

Since the carbamate viscosity is much higher than the viscosity of MEA (see the Supplementary Materials), a molecular rotor/amine solution spatially resolves the regions of increased carbamate concentration in the amine in real time. A molecular rotor/amine solution was prepared by adding 0.9 mg of CCVJ to 1.5 g of MEA and stirred for 16 hours with the vial wrapped in tin foil to avoid any light exposure. A device to hold the microcapillary, henceforth referred to as the holder, is outfitted with a thermocouple to read the temperature inside the holder and near the outer surface of the microcapillary. The holder was conditioned by flowing N_2_ for 30 min at 20 SCCM to purge all air. A microcapillary with the interior surface coated with octadecyltrichlorosilane was filled three-fourths of the way with the MEA/CCVJ solution through capillary action. The filled microcapillary was immediately placed in the N_2_-purged holder. To ensure that the microcapillary was centered and flat against the bottom surface of the holder, two cubes of quartz were glued to the interior sides of the stage, leaving a gap between the two just wide enough for the microcapillary to sit. Care was taken to ensure the quartz pieces did not block the inlet or outlet of the holder. The open end of the holder was then capped and wrapped in Teflon tape to ensure a watertight seal. The capped holder was placed into the bottom of a water bath sitting on the stage of an inverted fluorescence microscope (Nikon Eclipse Ti) while continuing to be purged with N_2_. The water bath temperature was set to 35°C. Once equilibrium was reached between the water bath temperature and the interior temperature of the holder, an image recording sequence was set up through the NIS Elements Advanced Research software to record images every 5 s under a GFP (green fluorescent protein) fluorescent filter set at 60-ms exposure. During the first 2 min of the image recording sequence, the N_2_-only flow continued. The remaining 28 min was a flow of CO_2_/N2 (1:1) at 20 SCCM. At the end of the experiment, the flow was returned back to N_2_ only and the water bath was quickly cooled to room temperature by removing the warmer water and adding cold water to the bath. An image was taken once the water bath reached room temperature. This entire procedure was repeated with fresh microcapillaries at 55° and 75°C.

### Dynamic spreading and contact angle experiments

A Nikon Eclipse Ti Inverted fluorescence microscope was used to measure the spreading velocity of a water droplet on the surface of a textured, 3D-printed AlSi_10_Mg filament. The filament was printed with the same parameters as the interior of the 3D-printed monolith used for the CO_2_ capture experiments. To mitigate any false spreading effects of water on glass, the filament was elevated above a microscope slide on pillars fabricated with stacked coverslips (gray cubes in fig. S6A).

To determine the effect of surface energy on the dynamic spreading, three experiments were run. In the first experiment, a filament was rinsed with ethanol, acetone, and dried with nitrogen. In the second experiment, a filament was cleaned using the same procedure previously mentioned and placed into a 400°C air oven for 3 hours to remove any carbon contamination from the surface and thereby increase the surface energy. In the third experiment, a filament was cleaned and plasma treated for 5 min in an air plasma chamber. The static contact angle of water was measured on representative smooth aluminum substrates subjected to the same surface treatments. The native aluminum substrate displayed a contact angle of 40° ± 2°. When plasma treated, the contact angle decreased to 18° ± 7°. Last, when oven treated in air at 400°C for 3 hours, the contact angle was not measurable, indicating that the spreading coefficient on this substrate was ≥0.

In all spreading experiments, the filament was placed on the center of the coverslip pillars and oriented so the left end of the filament was slightly before the edge of the pillar. Under a GFP fluorescent filter at 10-ms exposure, a 10-μl droplet of water + fluorescein (blue circle) was placed on the edge of the left side of the filament. The NIS Elements Advanced Research software was used to record this video. Spreading velocity was obtained by tracking the progress of the wicking front using image analysis.

The rate of spreading of water on rough substrates scales with the contact angle as ~(cosθ−cosθ_c_), where θ_c_ is the critical hemiwicking contact angle dictated by the surface texture (*43*). From our measurements, we observe that this linear scaling holds for the three different surface treatments (fig. S6D), thereby verifying that the static contact angle measurements on the flat substrate are representative of the fiber chemistry. Furthermore, we note that in the final state of uptake in the oven-treated system, most of the asperities are covered as would be expected for *S* > 0, whereas in the untreated fiber, the tops of asperities are visible (fig. S6, B and C).

### Molecular simulations of CO_2_ capture in liquid amine

The temperature effect of increased CO_2_ absorption was studied in detail through atomistic simulations. We used a reactive MD method coupled with a grand canonical Monte Carlo (GCMC) scheme for CO_2_ molecules. A liquid slab of MEA was created in a simulation box with dimensions 40 × 40 × 130 Å^3^ with periodic boundary conditions in all directions. The liquid slab is about 70 to 80 Å thick (in *z*) and consists initially of 1000 MEA molecules, which, over time, get consumed in reactions with CO_2_, generating carbamate as a product. The rest of the simulation box volume is filled with CO_2_ gas at a pressure of 0.5 atm.

To ensure a constant gas pressure and an excess amount of CO_2_, we define a rectangular region centered between, and sufficiently far away from, the two MEA/gas interfaces. The center of the exchange region is 35 Å away from the liquid surface and is 30 Å thick in *z*. Molecular exchanges (insertion or deletion of CO_2_ molecules) take place in the region through MC moves. In this way, CO_2_ molecules are simulated in a grand canonical ensemble at a given chemical potential, corresponding to a gas pressure of *p*_0_ = 0.5 atm. The entire simulation setup is shown in fig. S12.

We used MAPS 4.3 by Scienomics-SAS (*44*) to generate initial configurations and to assign the AMBER Cornell forcefield (*45*). Electrostatic potential (ESP) calculations were carried out to assign partial charges to MEA, CO_2_, and the carbamate products, MEAH^+^ and MEACOO^−^. Density functional theory calculations were performed with NWChem 6.6 to compute the ESP charges (*46*). The B3LYP functional was used with 6-311++G(2d,2p) basis set. For these simulations, the MAPS platform was also used to create all necessary input parameter and coordinate files of single molecules. For MD simulations, we used LAMMPS MD (*47*). The initial configuration consists of 1000 MEA molecules only (no CO_2_) in the described slab geometry. After energy minimization, the liquid was further equilibrated for 500,000 steps at 0.1-fs time steps with a constant box volume, *V*, and temperature, *T*. During this initial equilibration stage, the temperature was slowly ramped up from −173°C to its target temperature (32° and 71°C), which was maintained by a Nosé-Hoover thermostat with a 100–time step relaxation period. Long-range electrostatics were calculated with the particle-particle-particle-mesh method at a force accuracy of 10^−4^. In subsequent MD runs, now at 1-fs time steps, GCMC moves were invoked every 1000 MD steps, during which 100 attempts were made for insertion or deletion of CO_2_ molecules in the exchange region shown in fig. S12 at given chemical potential corresponding to the desired pressure of 0.5 atm. At this CO_2_ pressure, the ideal gas chemical potential value can be used for the GCMC moves.

Chemical reactions were modeled using a simplified reaction method developed recently by Gissinger *et al.* (*48*, *49*). The method is useful for known reaction pathways in which two atoms are bonded together once they get closer than a predefined distance. The reaction will be accepted at a given probability. In this study, reaction kinetics are solely controlled by these two parameters. One needs to adjust those parameters to obtain the desired reaction rates. More details of the MEA-CO_2_ reaction process model are given in the Supplementary Materials.

## Supplementary Materials

### This PDF file includes:

Supplementary Text Figs. S1 to S15 Table S1 Legends for movies S1 and S2 References
