# Supplementary material for: Solid with infused reactive liquid (SWIRL): A novel liquid-based separation approach for effective CO2 capture
Source: Sci Adv. 2022 Feb 9;8(6):eabm0144. doi: 10.1126/sciadv.abm0144 (PMC8827647; doi:10.1126/sciadv.abm0144)
Supplement: Supplementary file 1 — Supplementary Text Figs. S1 to S15 Table S1 Legends for movies S1 and S2 References [file sciadv.abm0144_sm.pdf]

Science Advances
AAAS

# Supplementary Materials for

## Solid with infused reactive liquid (SWIRL): A novel liquid-based separation approach for effective $CO_2$ capture

Mohsen S. Yeganeh*, Arben Jusufi, Shane P. Deighton, Matthew S. Ide, Michael Siskin, Aditya Jaishankar, Charles Maldarelli, Pedro Bertolini, Bharath Natarajan, Jessica L. Vreeland, Mark A. King, Andrew R. Konicek

*Corresponding author. Email: mohsen.s.yeganeh@exxonmobil.com

Published 9 February 2022, *Sci. Adv.* **8**, eabm0144 (2022)
DOI: 10.1126/sciadv.abm0144

**The PDF file includes:**

Supplementary Text
Figs. S1 to S15
Table S1
Legends for movies S1 and S2
References

**Other Supplementary Material for this manuscript includes the following:**

Movies S1 and S2

**Supplementary Text**

<u>Additive Manufacturing of Support Structure</u>

The design freedom enabled by additively manufactured techniques allows the facile synthesis of complex structures. While many possible geometric arrangements may fit the criteria of having a high surface area to volume, one must also consider the limits of manufacturing resolution, mechanical strength during and post-print, and the potential of the structure to produce porosity on the surface. Lattices offer an attractive solution due to the connectedness of the nodes and struts that maximize mechanical strength while still providing significant surface area. Due to the inherently small features, lattices are difficult and often impossible to fabricate through traditional manufacturing methods.

The direct metal laser sintering (DMLS) technique forms objects via the layer-wise successive deposition and binding of a metal powder using a laser as a heat source. The metal powder particle size distribution is chosen due to process flowability. A scanning electron microscope (SEM, Hitachi S-4800) image of the $AlSi_{10}Mg$ spherical particles is shown in Fig. S7. Controlling the energy density of the laser for the direct metal laser sintering process is important to ensure fabrication of the rough features at the surface. The laser energy density is a function of laser power, scan speed, and spot size. Traditionally, the process parameters for DMLS are chosen to maximize density and minimize surface roughness. This work used a modified set of process parameters in order to achieve a certain surface roughness.

An example of the significant effect processing parameters can have on the microstructure of a part is shown in SEM images (Fig. S1) for 800 μm $AlSi_{10}Mg$ cylinders (*i.e.* filaments) printed to

investigate the role of substrate curvature. The laser spot size was consistent at 0.08 mm. Fig. S1A was manufactured with a laser power of 350 W and scan speed of 1650 mm $s^{-1}$. SEM images of the part illustrate that it is relatively smooth with only a few rough features and little to no micro-porosity on the surface. The part shown in Fig. S1B was manufactured with a laser power of 250 W and scan speed of 1800 mm $s^{-1}$. The SEM images show a significant amount of rough features on the surface due to incomplete powder fusion. The rough features observed were firmly attached to the 3D printed structure but retain, to a variable extent, portions of their original spherical shape. While the surface texture is a function of both the processing parameters as well as the structure size and orientation, clearly, for a similar part, an order of magnitude difference in surface roughness can be achieved. The images show the roughness on the surface of the structure. However, the nodes (D) show more internal porosity, while the rods (E) show more external surface structure. The relationship between feature size and surface roughness for direct metal laser sintering is still being explored.

An additively-manufactured, finished, exposed support structure manufactured from $AlSi_{10}Mg$ is shown in Fig. S8A. The part was manufactured with a laser power of 250 W and scan speed of 1800 mm $s^{-1}$. The additively manufactured support structure manufactured from stainless steel 316 used a laser power of 44 W and scan speed of 1000 mm $s^{-1}$ (Fig. S8B). The objects were printed in a vertical orientation and were removed from the base plate after excess powder was removed from the part internals.

<u>General thermodynamic criteria for liquid immobilization on textured surfaces</u>

Consider an arbitrarily rough surface of actual area, $A_r$. The surface when projected onto a 2D plane has a projected area, $A_p$. There are three possible states of liquid immobilization on this surface (Fig. S9a-c):

1) The surface is non-wetting to the liquid of choice. In this *dry* state, the energy of the system is

$$E_{\mathrm{d}} = A_r \gamma_{sv} \tag{1}$$

where $\gamma_{sv}$ is the solid-vapor surface energy.

2) The liquid is immobilized in the texture, but only fills to the brim. In this *infused* state, the energy of the system is

$$E_{\mathrm{i}} = A_{sl}^{(i)} \gamma_{sl} + A_{sv}^{(i)} \gamma_{sv} + A_{lv}^{(i)} \gamma_{lv} \tag{2}$$

where $\gamma_{sl}$ and $\gamma_{lv}$ are the solid-liquid and liquid-vapor surface energies, respectively. $A_{sl}^{(i)}$, $A_{sv}^{(i)}$ and $A_{lv}^{(i)}$ are the solid-liquid, solid-vapor and liquid-vapor surface energies in the infused state, respectively.

3) The liquid is immobilized in the texture and also fully encapsulates it. In the *encapsulated* state, the energy of the system is

$$E_{\mathrm{e}} = A_r\gamma_{sl} + A_p\gamma_{lv} \tag{3}$$

Firstly, for the infused scenario to be energetically favored over the dry state, the following criterion must be satisfied

$$E_{\mathrm{i}} - E_{\mathrm{d}} < 0 \tag{4}$$

Substituting (1) and (2) into (4)

$$A_{sl}^{(i)}\gamma_{sl} + (A_{sv}^{(i)} - A_r)\gamma_{sv} + A_{lv}^{(i)}\gamma_{lv} < 0 \tag{5}$$

Since $A_r = A_{sv}^{(i)} + A_{sl}^{(i)}$, we get

$$A_{sl}^{(i)}(\gamma_{sl} - \gamma_{sv}) + A_{lv}^{(i)}\gamma_{lv} < 0 \tag{6}$$

Then if the spreading coefficient, *S*, is defined as

$$S = \gamma_{sv} - \gamma_{sl} - \gamma_{lv} \tag{7}$$

Equation (6) can be rewritten as

$$A_{sl}^{(i)}(-S - \gamma_{lv}) + A_{lv}^{(i)}\gamma_{lv} < 0 \tag{8}$$

or

$$S > \left(\frac{A_{lv}^{(i)}}{A_{sl}^{(i)}} - 1\right)\gamma_{lv} \equiv S_{i-d} \tag{9}$$

Equation (9) identifies a critical spreading coefficient ($S_{i-d}$) above which the infused state is preferred. The value of $S_{i-d}$ is dictated purely by the texture geometry and the liquid-vapor surface energy. We note that since $A_{lv}^{(i)} < A_{sl}^{(i)}$ (Fig. S9d), $S_{i-d}$ has a negative value.

Now, for the encapsulated scenario to be energetically favored over the infused state, the following criterion must be satisfied

$$E_{\mathrm{e}} - E_{\mathrm{i}} < 0 \tag{10}$$

Substituting (2) and (3) into (10)

$$\left(A_r - A_{sl}^{(i)}\right)\gamma_{sl} + \left(A_p - A_{lv}^{(i)}\right)\gamma_{lv} - A_{sv}^{(i)}\gamma_{sv} < 0 \tag{11}$$

Since $A_r = A_{sv}^{(i)} + A_{sl}^{(i)}$, we get

$$A_{sv}^{(i)}(\gamma_{sl} - \gamma_{sv}) + (A_p - A_{lv}^{(i)})\gamma_{lv} < 0 \tag{12}$$

Using (7), Equation (12) can be rewritten as

$$A_{sv}^{(i)}(-S - \gamma_{lv}) + (A_p - A_{lv}^{(i)})\gamma_{lv} < 0 \tag{13}$$

or

$$S > \left(\frac{A_p - A_{lv}^{(i)}}{A_{sv}^{(i)}} - 1\right)\gamma_{lv} \equiv S_{e-i} \tag{14}$$

Equation (14) identifies a critical spreading coefficient ($S_{e-i}$) above which the encapsulated state is preferred. The value of $S_{e-i}$ is dictated by the texture geometry and the liquid-vapor surface energy. Now we identify conditions for $S_{e-i}$ to be greater than $S_{i-d}$. From (9) and (14) we have

$$\frac{A_p - A_{lv}^{(i)}}{A_{sv}^{(i)}} > \frac{A_{lv}^{(i)}}{A_{sl}^{(i)}} \quad (15)$$

Rearranging we get

$$\frac{A_{sl}^{(i)}}{A_{lv}^{(i)}} > \frac{A_r}{A_p} \quad (16)$$

Let $A_b$, $A_w$, and $A_t$ be the areas of the bottom, side-walls, and top of the asperity, respectively. Therefore $A_r = A_b + A_w + A_t$, $A_{sl}^{(i)} = A_b + A_w$, and $A_{lv}^{(i)} = A_p - A_t$.

From Fig. S9, (16) can be expressed

$$\frac{A_b + A_w}{A_p - A_t} > \frac{A_b + A_w + A_t}{A_p} \quad (17)$$

$$(A_b + A_w)A_p > (A_b + A_w + A_t)(A_p - A_t) \quad (18)$$

$$A_r > A_p \quad (19)$$

Since $A_r > A_p$, the inequality in (19) is always true and therefore $S_{e-i}$ is always greater than $S_{i-d}$. This result suggests that if $S_{e-i}$ is surpassed by the chosen liquid-solid system or via surface modification, liquid immobilization is guaranteed. Further, as the dry top of the surface

in the infused state approaches a flat shape, as in microcapillary arrays, $(A_p - A_{lv}^{(i)}) \rightarrow A_{sv}^{(i)}$ and $S_{e-i}$ tends to 0 (Equation 14). This indicates that identifying or modifying substrates to have S>0 ensures successful immobilization.

Role of substrate curvature

Since the substrates in our study are filaments (*i.e.* cylindrical), we also deem it important to investigate the effect of substrate curvature on the wetting criteria. The curvature of the substrate imposes two key differences

(i) In the infused state, the sum of the solid-vapor and liquid-vapor interfacial area is greater than the projected area, which is the surface are of the basal cylinder ($A_p < A_{sv}^{(i)} + A_{lv}^{(i)}$), see Fig. S10a.

(ii) Similarly, in the encapsulated state the liquid vapor interfacial area is greater than the projected area ($A_p < A_{lv}^{(e)}$), see Fig. S10b.

Due to the larger liquid vapor interfacial area, there is an additional penalty imposed for wetting. As a result, the critical spreading coefficients $S_{i-d}$ and $S_{e-i}$ have larger values than their flat substrate counterparts (Equations (9) and (14)). However, the magnitude of this difference is dependent on the substrate curvature relative to the local curvature imposed by the microstructure.

To illustrate this point in quantitative terms we pick a grooved texture of lattice spacing *w*, wall thickness *t* and height *h* (see Fig. S10c). The curved substrate is a fiber of radius *b*. The derivation of the wetting criteria for the dry to infused transition on the fiber is identical to the flat substrate. For the grooved substrate the critical spreading coefficient on a flat substrate (of unit depth into the page) is

$$S_{i-d,flat} = \left(\frac{A_{lv}^{(i)}}{A_{sl}^{(i)}} - 1\right)\gamma_{lv} = \left(\frac{(w-t)}{(w-t)+2h} - 1\right)\gamma_{lv} \tag{20}$$

For the grooved substrate the critical spreading coefficient on a fiber of radius *b* is

$$S_{i-d,fiber} = \left(\frac{A_{lv}^{(i)}}{A_{sl}^{(i)}} - 1\right)\gamma_{lv} = \left(\frac{(w-t)(b+h)}{h^2 + 2bh + (w-t)b} - 1\right)\gamma_{lv} \tag{21}$$

The difference between these terms is plotted in Fig. S10d for a range of *w* and *b* values at $t$ = 20 µm and $h$ = 30 µm. As is evident, $S_{i-d,fiber} > S_{i-d,flat}$ and the difference becomes larger when the fiber radius approaches the asperity size. We note that while $S_{i-d,fiber} - S_{i-d,flat}$ is positive, both critical spreading coefficients are negative numbers.

For the infused to encapsulated transition, we note, comparing to Equation (3), $A_{lv}^{(e)} \neq A_p$. Barring this difference, the rest of the derivation is identical. For the grooved substrate the critical spreading coefficient on a flat substrate (of unit depth into the page) is

$$S_{e-i,flat} = \left(\frac{A_p - A_{lv}^{(i)}}{A_{sv}^{(i)}} - 1\right)\gamma_{lv} = 0 \tag{22}$$

For the grooved substrate the critical spreading coefficient on a fiber of radius *b* is

$$S_{e-i,fiber} = \left(\frac{A_{lv}^{(e)} - A_{lv}^{(i)}}{A_{sv}^{(i)}} - 1\right)\gamma_{lv} = \left(\frac{w(b+h+e) - (w-t)(b+h)}{(b+h)t} - 1\right)\gamma_{lv} \quad (23)$$

where $e$ is the thickness of the encapsulating liquid film. The difference between these terms is plotted in Fig. S10e for a range of w and b values at e = 1 µm, t = 20 µm and h = 30 µm, for water ($\gamma_{lv}$=72.8 mN/m). As is evident, $S_{e-i,fiber}$ > $S_{e-i,flat}$ = 0. However, this difference is only significant when the fiber radius approaches the asperity size. Since our fiber is significantly larger than the asperity size, we believe our approach to making S>0 is sufficient for the 3D printed filaments. We pick a grooved structure because here, the fiber's curvature is imposed on the entire liquid volume, which represents an extreme case. In a substrate with an arbitrarily rough surface, the local curvature will likely have a stronger effect than the underlying fiber curvature, which could relax the wetting criterion back to a flat surface case.

Modeling the diffusion of carbamate

We model the absorption of the $CO_2$ in the amine as a pure diffusion problem. As outlined in the main text of the paper, the reaction between $CO_2$ with the amine is nearly instantaneous and occurs kinetically with a high Damköhler number (*i.e.* the rate of the chemical reaction is much faster than the rate of diffusion). This is supported by the references noted in the main paper. Therefore, a very good approximation for this reaction-diffusion process is to assume that the chemical reactions that generate the carbamate occur right at the interface between the amine and the $CO_2$, and this generated carbamate then diffuses downward into the bulk amine. This means that there are few to no $CO_2$ molecules in the bulk amine, having all been consumed at the surface, and no further reactions taking place in the bulk. Under this picture, we can simplify the problem to that of pure diffusion of carbamate generated at the interface into the bulk amine.

Under the conditions of the experiments conducted in the capillary tube (see Fig. 3A-B of the main paper) and over the timescale of the experiment the capillary tube can effectively be considered to be infinitely long. In other words, the diffusion process is so slow that the carbamate effectively never sees the end of the capillary tube. This assumption is supported by the fact that the Damköhler number is of order $10^4$ making diffusion much smaller than the reaction.

Consider a semi-infinite domain with pure diffusion occurring from the surface. At the surface of the domain the $CO_2$ concentration is $c(0,t) = c_0$ for all times. We are interested in finding the evolution of the concentration $c(x,t)$ below this surface as a function of both $x$ and $t$. The unsteady diffusion equation is $\frac{\partial c}{\partial t} = D\frac{\partial^2 c}{\partial x^2}$, where $c$ is the concentration of the carbamate in the bulk amine, and $D$ is the diffusion coefficient. The boundary conditions are $c(0,t) = c_0$ and $c(x,t) \to 0$ as $x \to \infty$, and the initial condition is that there is no carbamate in the bulk at $t = 0$, and hence $c(x,0) = 0$ for $x > 0$. This equation along with these boundary conditions is easily solved using the Laplace Transform Technique, and the solution is

$$c(x,t) = c_0 \mathrm{erfc}\left(\frac{x}{2\sqrt{Dt}}\right)$$

where erfc denotes the complementary error function. Details of the derivation of this solution can be found in standard mass transport textbooks (*55*). Because the molecular rotor experiments give us fluorescence intensity as a function of position (not concentration directly), to compare the above model with experiments we first convert the concentration expression to an intensity expression. To do this we first use the fact that the fluorescence intensity $I$ of a molecular rotor is related to the viscosity $\eta$ of its surrounding through the following relationship (*56*):

$$I = \alpha\eta^{\beta} \tag{22}$$

where $\alpha$ and $\beta$ are constants that depend on the specific dye used and the optical setup of the experiment. To relate the viscosity and concentration, there are various empirical laws that dictate the viscosity of blend of components through mixing rules. Here, we choose the Kendall and Monroe relationship that states that (*57*)

$$\eta^{1/3} = \phi\eta_A^{1/3} + (1-\phi)\eta_B^{1/3} \tag{13}$$

where $\eta, \eta_A, \eta_B$ are the viscosities of the mixture, component A, and component B, respectively, and $\phi$ is the mole fraction of component A. In our notation, species A is the carbamate and species B is the amine. Although Eq. (13) states that the viscosity of the mixture (and hence the diffusion coefficient) is a function of the concentration, in the above analysis solving for $c(x,t)$, we have made the approximation that this dependence on concentration is small, given the 1/3 power dependence. This implies that the diffusion coefficient is assumed to be a constant in the differential equation. To be more accurate we should solve the full non-linear equation accounting for the spatially varying diffusion coefficient, which is beyond the scope of this paper. However, as we show below, our approximate model captures the measured data closely. Substituting Eq. (13) into Eq. (22), and noting that the mole fraction $\phi = c(x,t)$ (assuming the units of concentration is mole fraction), we have

$$I = \left[C_1 erfc\left(\frac{x}{2\sqrt{Dt}}\right) + C_2\left(1 - erfc\left(\frac{x}{2\sqrt{Dt}}\right)\right)\right]^{3\beta} \tag{24}$$

Here $C_1 = \alpha\eta_A^{1/3}$, $C_2 = \alpha\eta_B^{1/3}$, and $\beta$ are fitting constants. Because species A is the carbamate, which is much more viscous than species B (the amine), we expect to have $C_1 > C_2$. To show the role played by the constant $\beta$, in Fig. S11 we choose different values of $\beta$ and plot the normalized fluorescent intensity

$$\bar{I} = \frac{I(x,t) - I_\infty}{I_0 - I_\infty} \tag{25}$$

as a function of distance from the interface $x$, where $I(x,t)$ is given by Eq. (24), $I_0$ is the intensity at $x = 0$ and $I_\infty$ is the intensity far away from the interface (which arises from the viscosity of the pure amine). The other parameters chosen for this example are given in the figure caption. We see that $\beta$ can cause a variation in the shape of the curve, and the curve does not necessarily look like a Gaussian (or error function).

To test the results of Eq. (24) against data, we run the following experiment: We first fill a capillary tube with amine mixed with a small concentration of molecular rotor. At time $t = 0$, the tube is exposed to $CO_2$ at a fixed temperature, leading to the interaction of $CO_2$ with amine to generate carbamate. This carbamate has a higher viscosity than the amine, and hence the molecular rotor displays a larger fluorescent intensity. In Fig. 3C we show the result of measuring the fluorescent intensity along the tube, at three different temperatures, after the absorption process has proceeded for $t = 28$ minutes.

The symbols correspond to data collected at different temperatures, while the black lines are fits of Eq. (24) to the data. While performing the fits, we held $\beta$ = 0.75 which was obtained from an

independent experiment that measured the viscosity of the amine-molecular rotor mixture at various temperatures against the corresponding fluorescent intensity at those temperatures. The fitting parameters were the diffusion coefficient $D$ and the ratio of the viscosity of the carbamate to the amine $\eta_A/\eta_B$. In all cases the fits of Eq. (24) to the data are good, signifying that the simple model of assuming all carbamate generation occurs at the gas liquid interface is a good physical model for the more complicated process of reaction-diffusion. The values of the diffusion coefficient and the ratio of the carbamate viscosity to the amine viscosity is presented in Table S1. We note that the diffusion coefficient increases with temperature, because viscosity decreases with increasing temperature which speeds up diffusion. This also leads to our experimental observation of increasing absorption capacity with increasing temperature at a fixed time of absorption.

These diffusion coefficients are in the correct range of values that we would estimate from the Stokes-Einstein relationship. This relationship states that

$$D = \frac{k_B T}{6\pi\eta r} \tag{26}$$

where $k_B$ is the Boltzmann constant, $T$ is the temperature, $\eta$ is the viscosity of the medium, and $r$ is the molecular size of the diffusing species. Using the values of $T = 300$ K, $\eta = 0.01$ Pa s, $r =$ 1 nm, we find that $D = 2.2 \times 10^{-11}$ $m^2s^{-1}$. This estimated value is close to the values measured through the above analysis shown in Table S1.

Reactive Molecular Dynamics of $CO_2$ in liquid amine

Before we provide details of the MEA-$CO_2$ reaction process we need to ensure that our molecular modeling approach simulates the same mass transport mechanism as observed experimentally. As discussed above the mass transport is determined by the balance of diffusion and reaction rates, quantified by the Damköhler number $Da = \frac{k_r}{D_0} L^2$, with $k_r$ being the reaction rate in $s^{-1}$, $D_0$ being the diffusion coefficient of reacting species, and $L$ denoting a lengthscale over which the reaction takes place. The real system has an amine layer thickness of $L \approx 50$ μm and the diffusion coefficient is on the order of $10^{-11}$ $m^2/s$ or smaller (see above). Using a reaction rate of $k_r = 100$ $s^{-1}$ we obtain a Damköhler number $Da \approx 10^4$ or larger. Our molecular simulation system needs to have a similar value to ensure the same diffusion-reaction balance. Since the diffusion coefficient cannot be varied in atomistic simulations (it is a system property solely based on the forcefield used), and the layer thickness is about 4 nm (half the liquid slab) we need to set the reaction rate to $k_r \approx 10^7$ $s^{-1} = 100$ $ns^{-1}$. The value does not need to be exact and is rather a guidance on how to choose the parameters in the reactive MD model to ensure that we can observe the reaction-diffusion mechanism on the molecular scale.

For MEA-$CO_2$ reactions the following two-step reaction scheme was implemented (*58*),

$$MEA + CO_2 \rightarrow MEACOO^- + H^+$$

$$MEA + H^+ \rightarrow MEAH^+$$

The overall product is carbamate consisting of the anion $MEACOO^-$ , $[OH\text{-}(CH_2)_2\text{-}NH\text{-}COO]^-$, and the ammonium cation $MEAH^+$, $[OH\text{-}(CH_2)_2\text{-}NH_3]^+$. The first reaction step above is modeled as follows: If during the simulation the distance between the nitrogen atom of an MEA molecule

and the carbon atom of a $CO_2$ molecule is within a predefined distance of 3.4 Å, a reaction is attempted and will be accepted with a probability of 0.5%. Once a reaction is accepted, the two atoms (N and C) are bonded and a carbamate anion generated. In this process a proton is released from the amine. In the second reaction step the released proton is bonded to another MEA if its nitrogen gets closer than 3.4 Å of the proton. Since this reaction is typically fast, the probability of accepting this bond was set to 100%. After each reaction a short energy minimization is performed to relax the system. With these settings we obtain reaction rates on the order of 100 $ns^{-1}$, as the above Damköhler number analysis requires. We ignore backward reactions since they are surpressed at high $CO_2$ pressure (here 0.5 atm).

The MEA-$CO_2$ reaction can now be observed on the molecular scale. Fig. S13 shows the number of carbamate molecules produced as the reaction progresses with time. With initially 1,000 MEA molecules, a maximum of 500 carbamate molecules can be produced theoretically in this system. The reaction rates are ~150 $ns^{-1}$ and are obtained from the initial slope of a linear fit of the curves within the first 0.5 ns at both shown temperatures. This value ensures a realistic Damköhler number. The reaction kinetic curves increase linearly in the beginning, however, the reaction significantly slows down after about 1 ns, depending on temperature. The maximum $CO_2$ absorption capacity is never reached during the simulation time of 10 ns in neither cases, 32 °C or 71 °C. There is, however, a temperature effect on the overall absorption capacity. Comparing “plateau” values at 10 ns, about 10% more MEA molecules are converted at 71 °C compared to the conversion at 32 °C. The difference - although not very high - is reproducible.

The mechanism for this behavior can be explained by higher diffusion and mixing of amine molecules as temperature is increased. Fig. S14 shows the density profiles of all species as a function of the *z* coordinate, denoting the value of the normal vector of the liquid/gas interface. Shown are results for 32 °C and 71 °C, both at a $CO_2$ partial pressure of 0.5 atm. In the beginning - the first 20 ps - only amine is present corresponding to the initial liquid slab thickness of around 70 - 80 Å. As time progresses carbamate is produced directly at the gas liquid interface. This is demonstrated by the occurrence of red peaks that have noticeably established within the first 0.5 ns. Those peaks are positioned at ~20 Å from the left interface and at ~80 Å from the right interface. At 71 °C, the peaks are slightly broader compared to the 32 °C case (at 0.5 ns). Juxtaposing the 2 ns data, one can clearly see that the carbamate peak has further broadened at higher temperature, indicating the carbamate diffused further into the bulk region. The inward diffusion of carbamate species entails an outward diffusion of bulk amine towards the interface, enabling more reactions with $CO_2$. We note that $CO_2$ molecules never reach the bulk amine region; in fact one can observe little enrichment of $CO_2$ in the carbamate interface layer at 2 ns (small green peaks). This is an important result since it reveals a detail of the reaction-diffusion mechanism: due to the fast reaction MEA is only consumed at the interface, and not in the bulk liquid. The increased consumption of MEA at elevated temperature is promoted due to higher diffusion of carbamate and amine, enabling an increased exchange of carbamate produced at the interface with fresh amine from the bulk.

<u>Pressure drop calculations</u>

Pressure drop is an important parameter in absorption bed design. The geometry selected as the SWIRL solid substrate not only provides a high A/V but also has a high void volume fraction. This is particularly important to minimize the pressure drop throughout the absorption bed.

To estimate the pressure drop that occurs as the gas flow is driven through the SWIRL device, we approximate the flow through the SWIRLS's hexagonal Laves structure as a flow through an ordered porous medium (Fig. S15). Several approaches can be used to describe such a gas flow through porous media (e.g. Karmen-Kozeny relations for flow through packed beds). Closer examination of the large scale (60x) mock-up model of the Laves structure shown in Fig. 1D, and also in Fig. S15, reveals that the gas flows mainly through parallel channels. From Fig. S15 the radius of these channels, approximated as circular cross section, is about R=0.5 mm at a volume fraction of approximately $\emptyset = 0.5$. For a superficial velocity of 1 m/s, the velocity through each channel is $\frac{1}{\emptyset} \equiv < V > =$2 m/s. The pressure drop per unit length, according to Poiseuille's Law, is $\frac{\Delta P}{L} = \frac{8\ \mu <V>}{R^2}$. Using the above values and a viscosity for air at 100 $^0$C , $\mu$ =2.7x10$^{-5}$ kg/(m s), we obtain $\frac{\Delta P}{L}$ =1728 Pa/m = 17 mbar /m. Poiseuille's flow is used because the Reynolds number for the gas flow is $\frac{\rho <V> 2R}{\mu}$= 70 (much smaller than the laminar flow limit of 2100) for a flow velocity in the channels equal to 2 m/s and a kinematic viscosity of air equal to 2.7 x10$^{-5}$ m$^2$/s. More complex approximations for gas flow through channels with hexagonal cross sections reveal similar pressure drops of below 20 mbar/m (*59*).

**Fig. S1.**

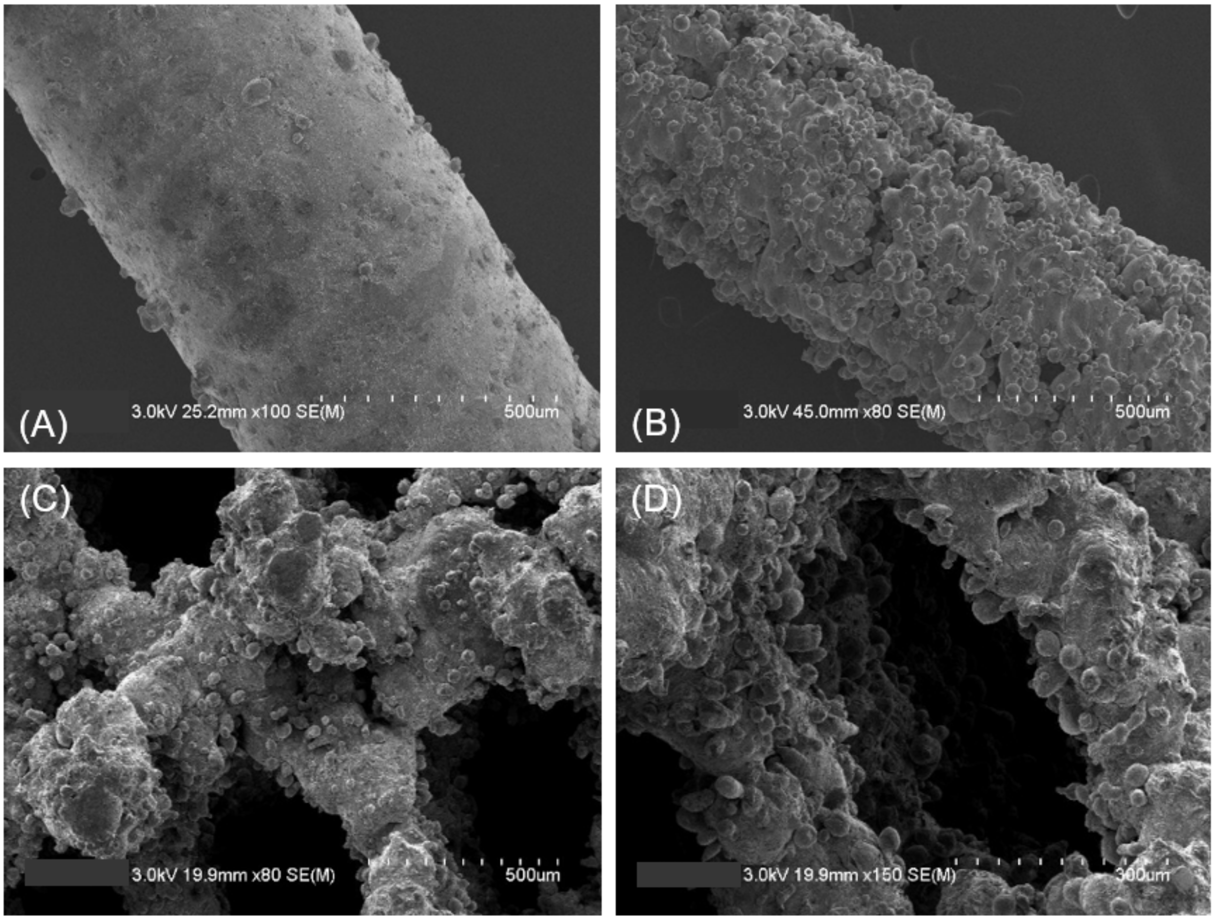


Fig. S1. **Microscopy images of solid surface roughness of SWIRL.** Additively manufactured $AlSi_{10}Mg$ 800 µm cylindrical parts manufactured with an energy density of: A) 2.7 J $mm^{-2}$ and B) 1.7 J $mm^{-2}$ illustrating the significant effect of processing parameters on the resultant surface texture. SEM images of C) the node and D) rods of the 3D printed exposed support hexagonal Laves structure, shown in Fig. 1B, manufactured with $AlSi_{10}Mg$ at an energy density of of 1.7 J $mm^{-2}$.

**Fig. S2.**

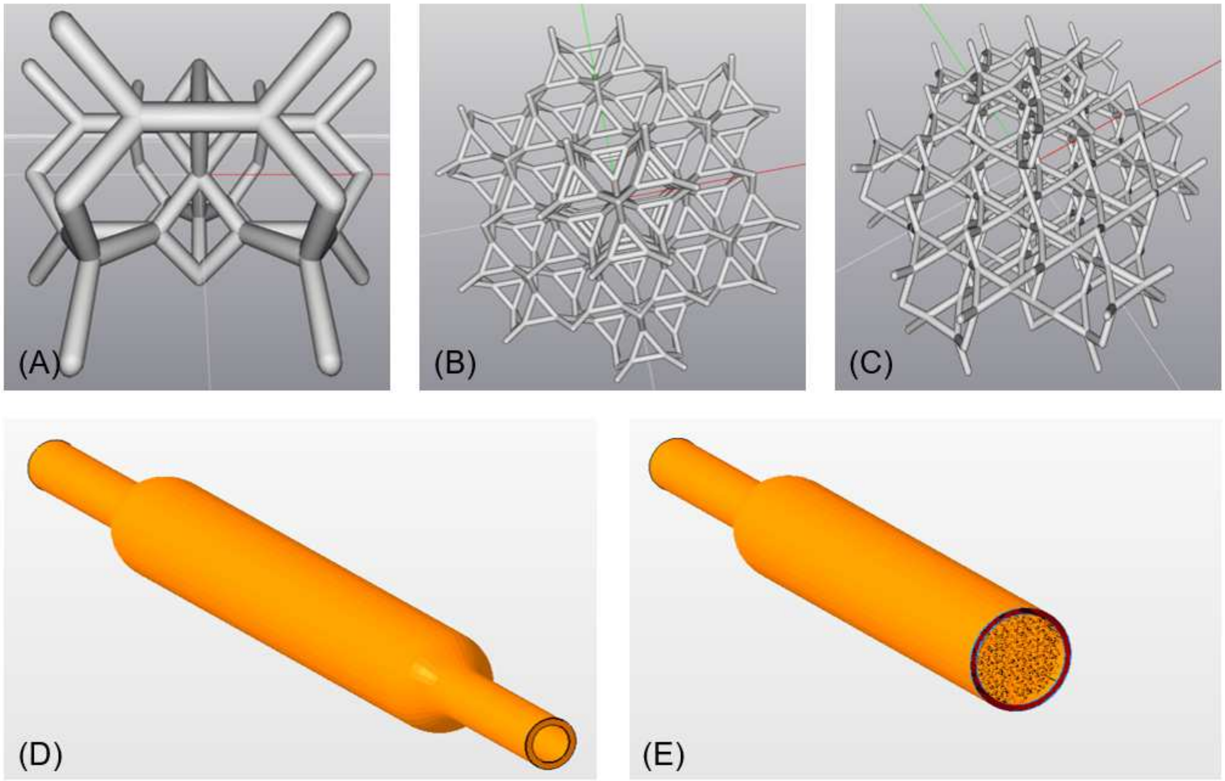


Fig. S2. **Graphics of 3D-printed Laves structures.** Node and strut skeleton for the hexagonal prism Laves phase drawn by nTopology shown in A) Unit cell side orientation, B) Top down for a multi-unit cell, and C) 45$^{o}$ orientation for a multi-unit cell. The designed support structure with an internal hex Laves phase lattice unit cell encompassed by a solid body drawn by Autodesk Netfabb Ultimate with views of the: D) The addition of the outer shell printed around the hexagonal Laves structure, providing a gas-tight tube for the absorption studies. E) Clipped tube in the xy-plane to show the internal lattice.

**Fig. S3.**

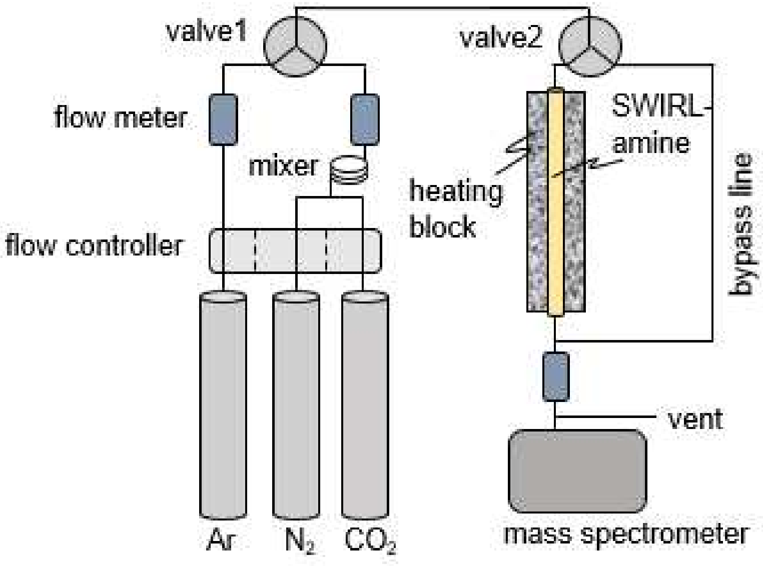


Fig. S3. **Experimental diagram for $CO_2$ capture using SWIRL.** Absorption unit. A preset ratio of $N_2$ and $CO_2$ are mixed before passing through an Omega mass flow meter. Valve 1 allows the flow of Ar or $CO_2/N_2$ mixture. Valve 2 enables the selected gas either bypass or flow through the SWIRL- amine before going through an Omega mass flow meter and the mass spectrometer.

**Fig. S4.**

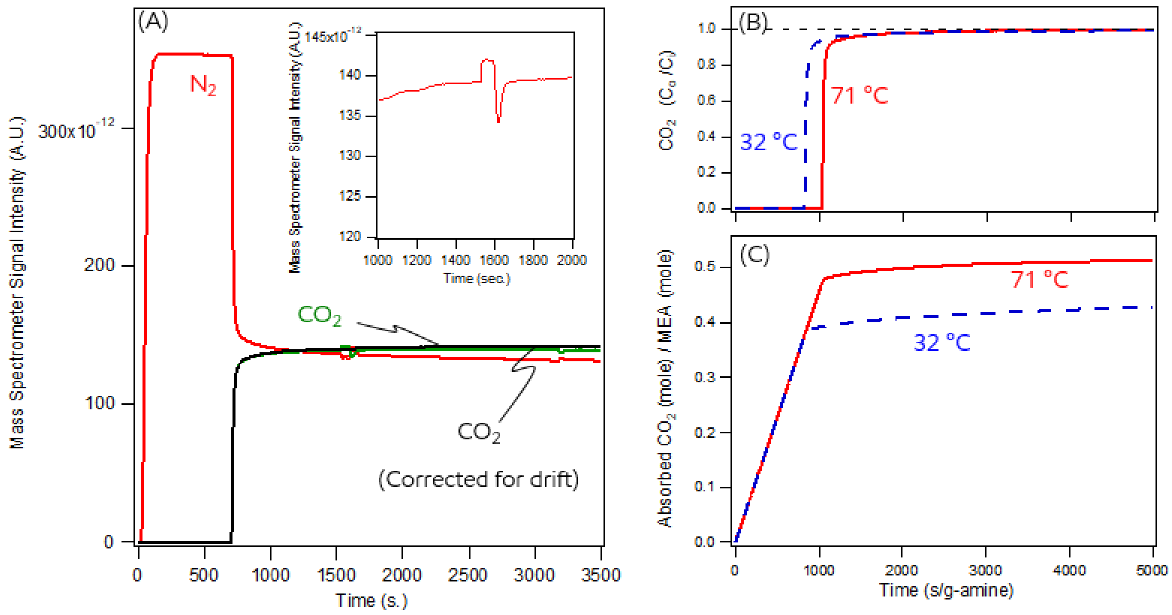


Fig. S4. **Breakthrough curve and absorption capacity profiles of SWIRL-MEA at different temperature.** A) Breakthrough curve for SWIRL-MEA at 71 °C that contains 0.6992 g of neat amine. The curves are raw mass spectrometer signal intensities and are not normalized. A mixture of $CO_2$/ $N_2$ gas is sent to SWIRL-MEA. When $N_2$ reaches the mass spectrometer time $t_0$ is recorded. While $N_2$ passing through SWIRL-MEA no $CO_2$ gas reaches the mass spectrometer up to about 704 seconds. At this time the $CO_2$ mass spectrometer intensity increases and the $N_2$ intensity drops. The drop in $N_2$ signal is due to the increase in the $CO_2$ partial pressure and thus a drop in partial pressure of $N_2$, which is detected by the mass spectrometer. After $CO_2$ passes the breakthrough point the mass spectrometer CO2 intensity increases and reaches the level of the input $CO_2$ concentration intensity. The bumps in the corresponding intensity curves shown at 1550 and 3100 sec. are the bypass signal intensities. The inset in Fig. S4A shows an enlargement

of the bypass signal intensity at 1550 sec. B) Normalized $CO_2$ signal as a function of time per gram of amine (sec/g-amine). C). Cumulative integrals of Fig. S4B curves, representing the amount of $CO_2$ absorbed/amount of amine used (mole per mole) as a function of time/g–amine.

**Fig. S5.**

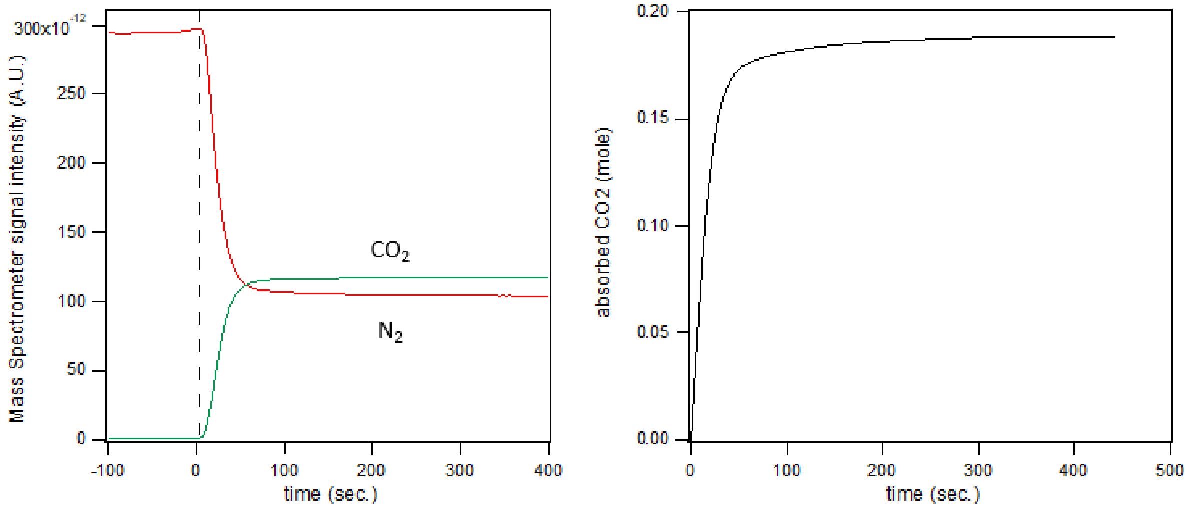


Fig. S5. **Breakthrough curve and capacity of empty cell.** A) Breakthrough curve for empty cell at 32 °C. Mass spectrometer signal intensity of $CO_2$ and $N_2$ as a function of time are shown. The curves are raw mass spectrometer signal intensities and are not normalized. $t_0$ is indicated by a dashed line. B) Capacity of the empty cell as function of time. The curve was produced through time integration of the breakthrough curve.

**Fig. S6.**

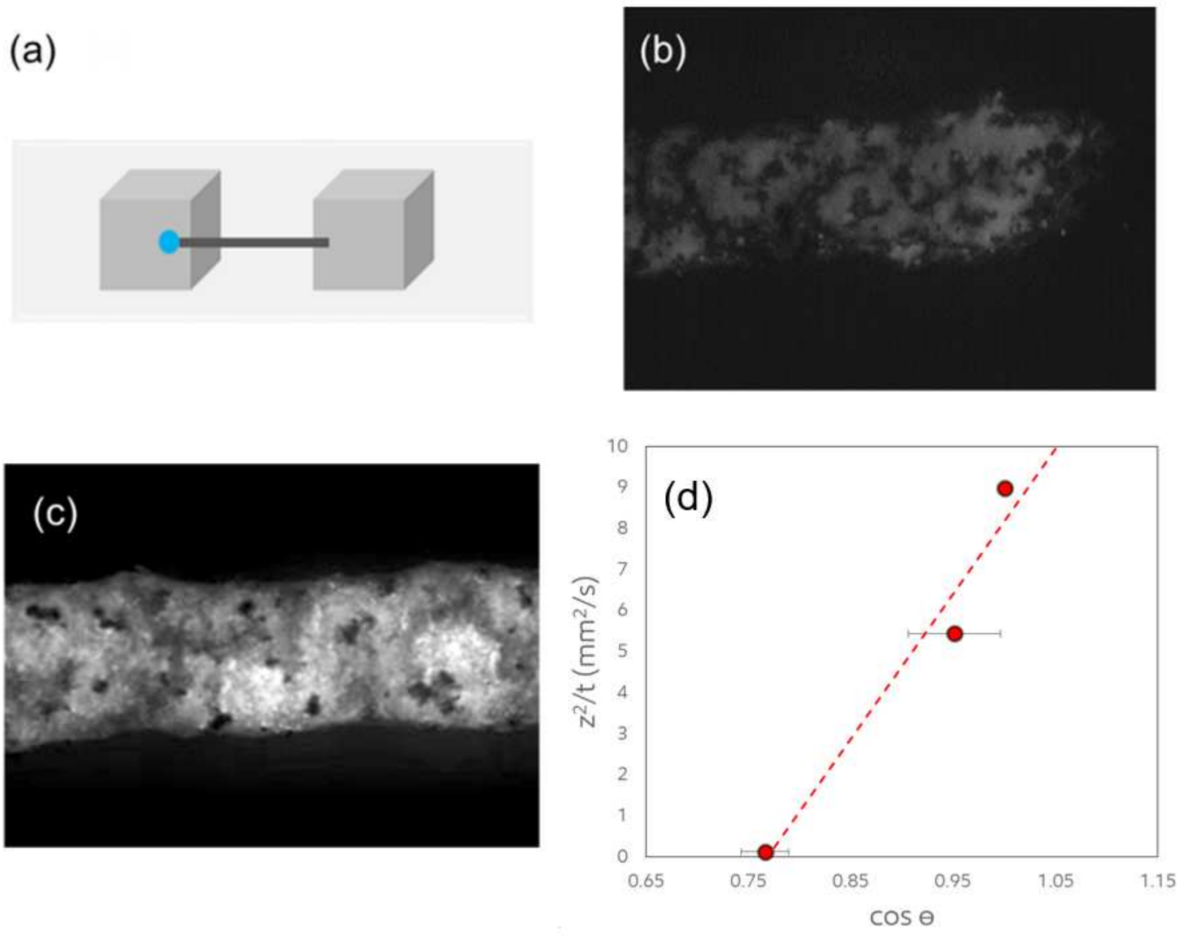


Fig. S6. **Liquid spreading on rough fiber surface.** (a) Schematic illustrating fiber and droplet placement in spreading experiments. Fluorescence microscopy images showing water wet surfaces of (b) native (S = -16 mN/m) and (c) oven-treated (S ≥ 0 mN/m) 3D-printed aluminum fiber surfaces. (d) Plot of spreading pre-factor as a function of the cosine of the equilibrium contact angle on a representative flat surface.

**Fig. S7.**

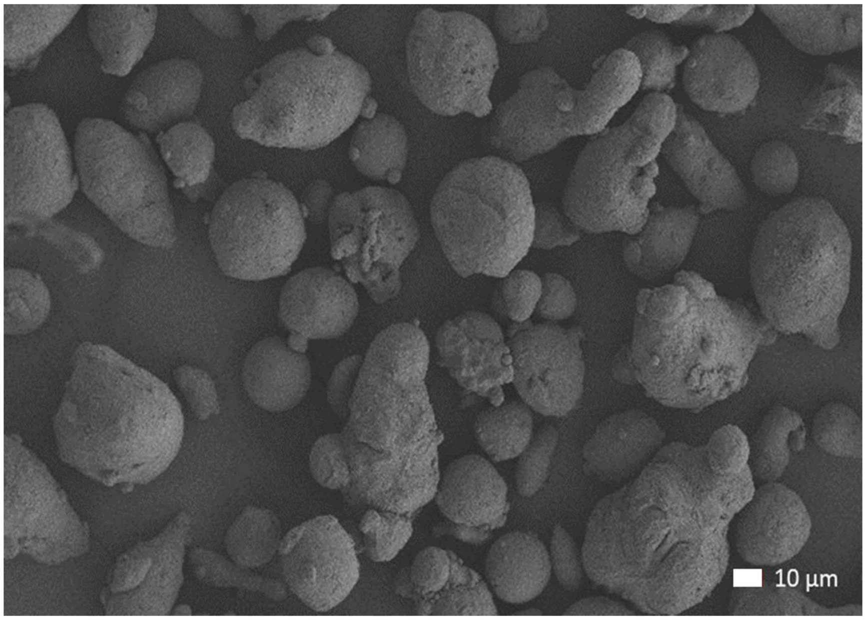


Fig. S7. **SEM image of AlSi10Mg spherical particles.**

**Fig. S8.**

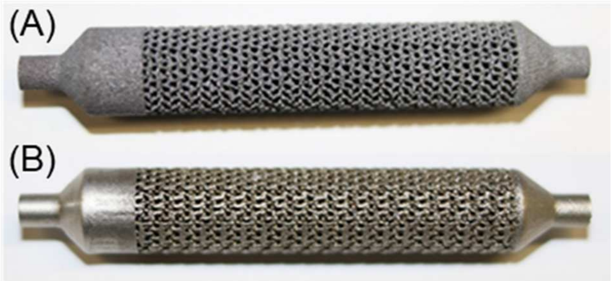


Fig. S8. **Images of additively manufactured support structures.** Exposed additively manufactured support structures composed of (A) $AlSi_{10}Mg$ and (B) SS316.

**Fig. S9.**

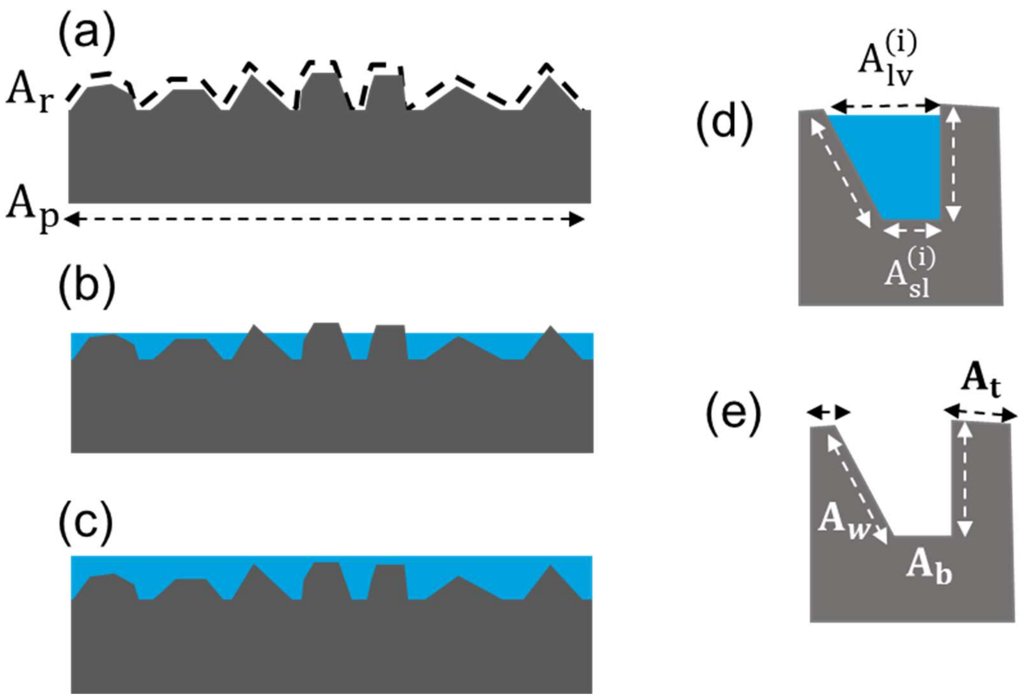


Fig. S9. **Schematic illustrations of wetting states on rough planar surfaces.** Schematic illustrating the dry (a), infused (b) and encapsulated states. (d) Schematic of representative asperity illustrating that $A_{lv}^{(i)}<A_{sl}^{(i)}$. (e) Schematic of representative asperity noting the top, side-wall and bottom areas.

**Fig. S10.**

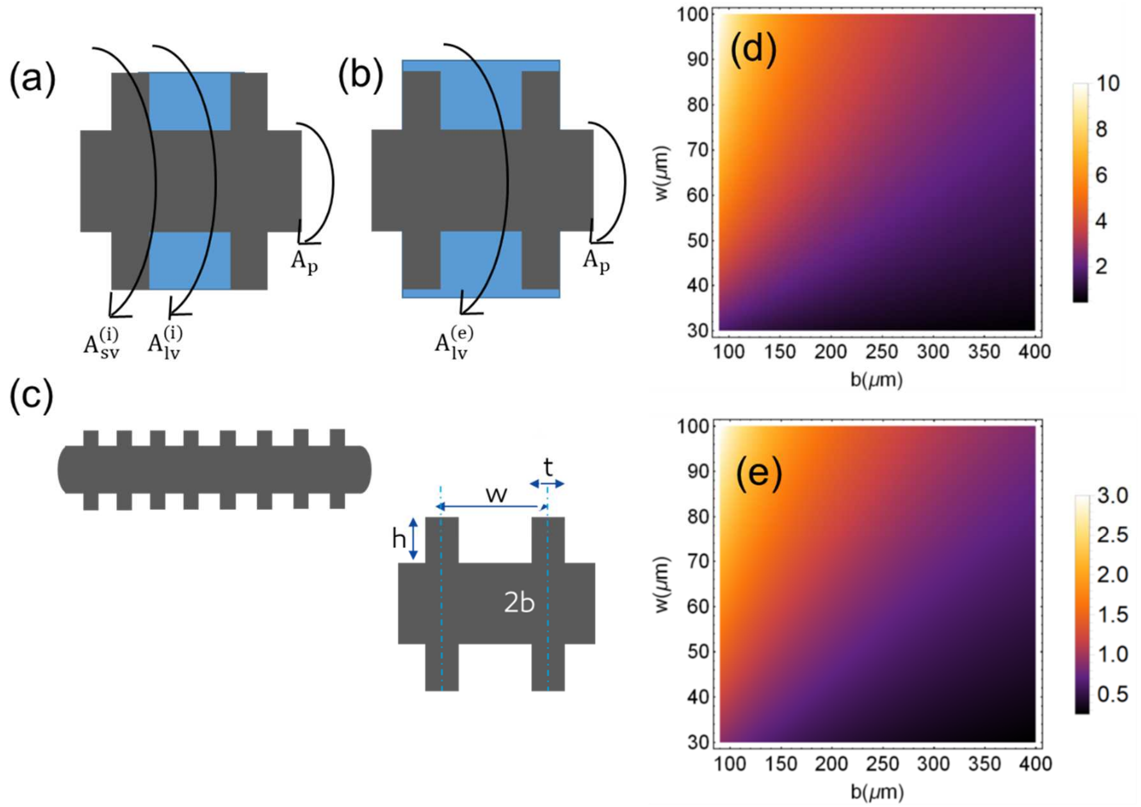


Fig. S10. **Wetting conditions on rough cylindrical fibers.** (a) Schematic illustrating the $A_p < A_{sv}^{(i)} + A_{lv}^{(i)}$ condition for a liquid infused on a fiber surface. (b) Schematic illustrating the $A_p < A_{lv}^{(e)}$ condition for a liquid encapsulated on a fiber surface. (c) Schematic of the model fiber used for energy calculations. (d) Density plot of $S_{i-d,fiber} - S_{i-d,flat}$ in units of mN/m for various $w$ and $b$ values. (e) Density plot of $S_{e-i,fiber} - S_{e-i,flat}$ in units of mN/m for various $w$ and $b$ values.

**Fig. S11.**

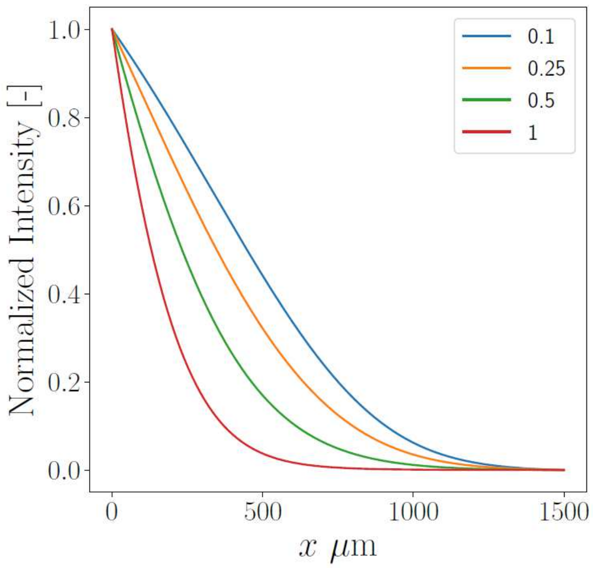


Fig. S11. **Influence of the exponent $\boldsymbol{\beta}$ on the shape of the intensity curves.**

**Fig. S12.**

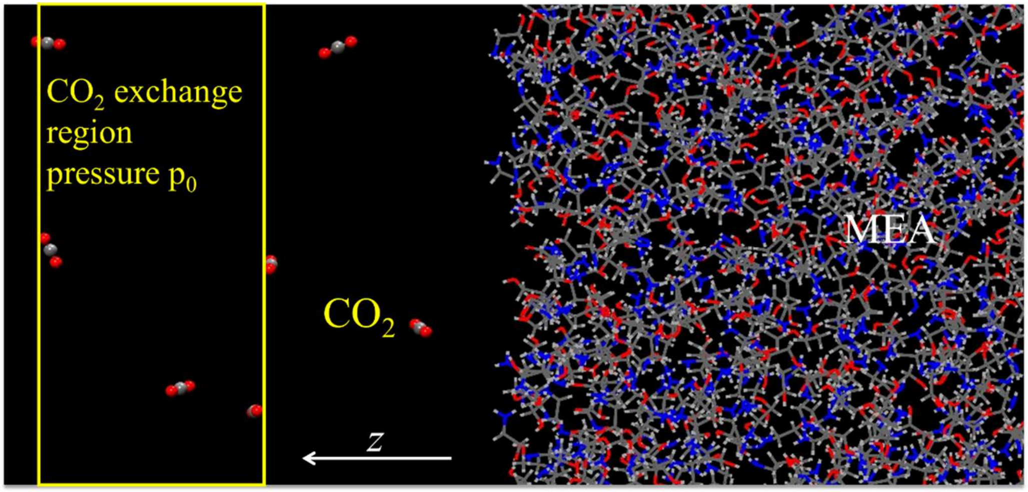


Fig. S12. **MD simulation snapshot of liquid amine interfacing a CO2 gas phase.** Simulation snapshot of liquid amine (MEA) slab interfacing with a $CO_2$ gas phase. The other interface - based on periodic boundary conditions - is not shown. The yellow box indicates the $CO_2$ molecule exchange region which is coupled to a virtual $CO_2$ reservoir to maintain the gas pressure, $p_0$, at the desired level in the region.

**Fig. S13.**

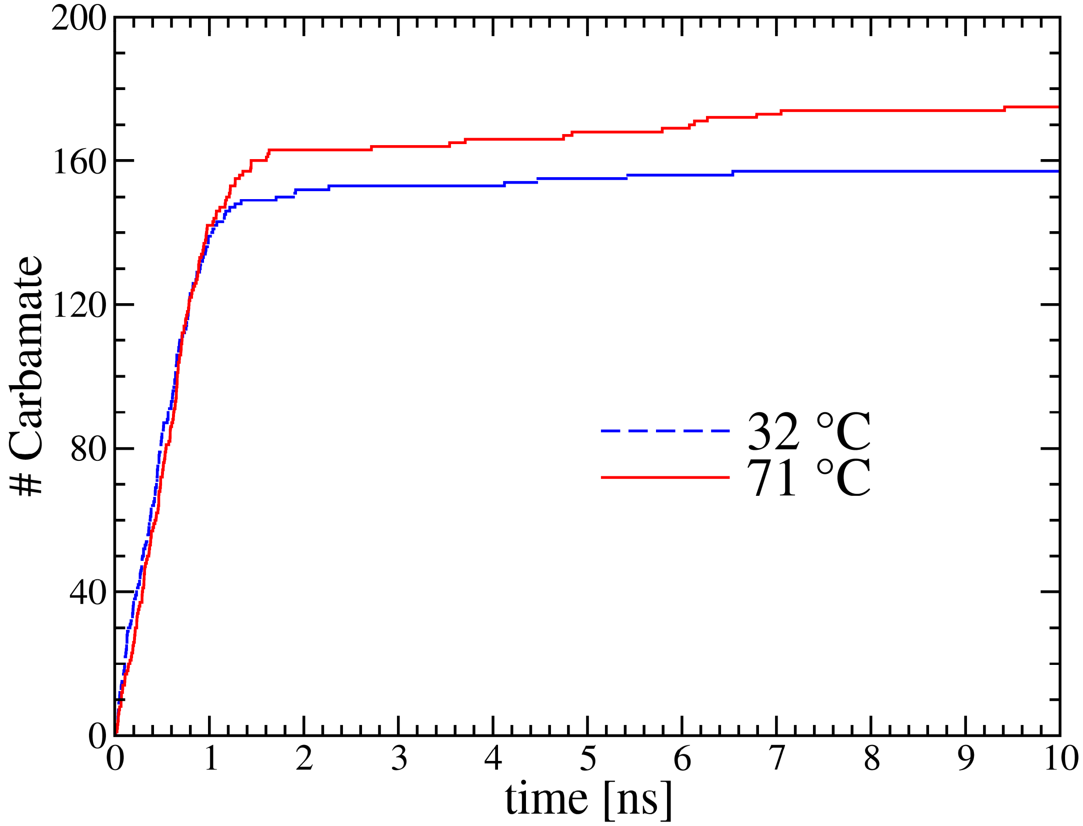


Fig. S13. **MD simulation results of carbamate formation time profile.** Number of carbamate molecules produced over time, obtained from molecular simulations of MEA and $CO_2$ performed at 32 °C and 71 °C. The $CO_2$ partial pressure was set to 0.5 atm. The maximum number of producible carbamate is 500.

**Fig. S14.**

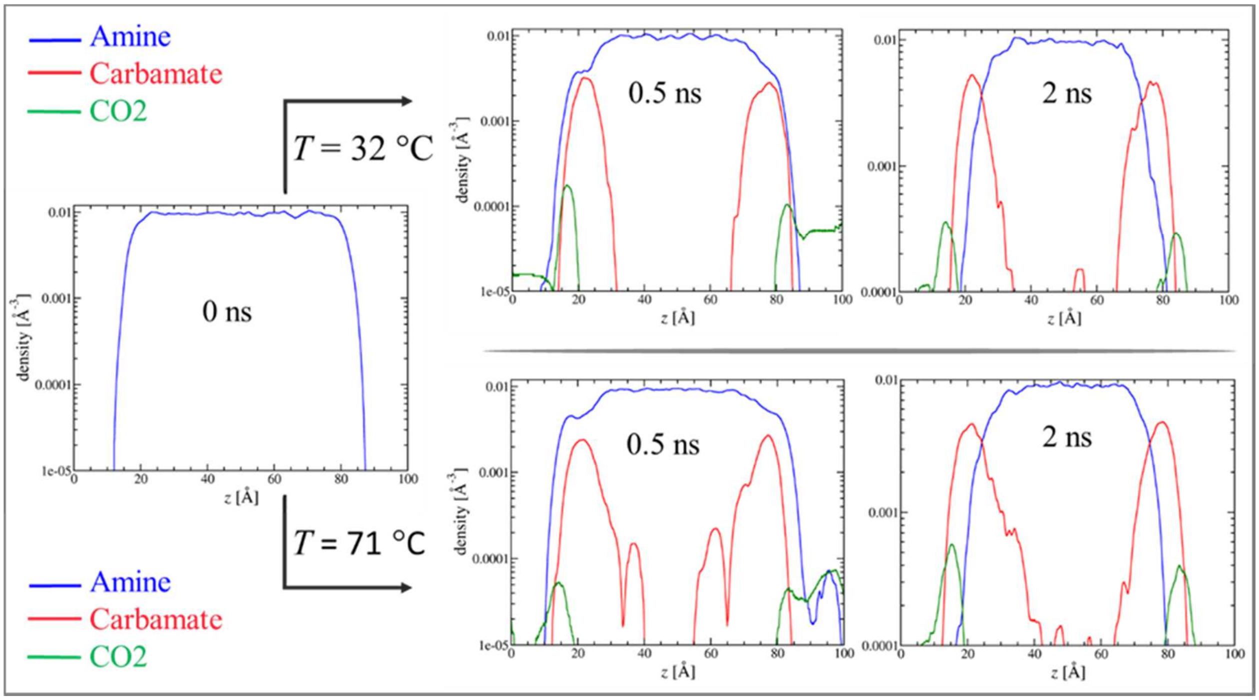


Fig. S14. **Changes of simulated molecular density profiles at different time and temperature.** Density profiles of chemical species, amine (MEA, blue lines), carbamate (MEAH-MEACOO, red lines), and $CO_2$ (green lines), along the normal interface coordinate *z*. Shown are profiles at different times (0 ns, 0.5 ns, and 2 ns) at 32 °C (top panel), and 71 °C (bottom panel), all at $CO_2$ partial pressure of 0.5 atm. The curves denote *z*-positions of nitrogen densities (representative atoms of amine or carbamate molecules) and of carbon densities (C in $CO_2$). The densities were averaged over 200 time frames (corresponding to 20 ps), and smoothened over 2 Å bin sizes along *z*.

**Fig. S15.**

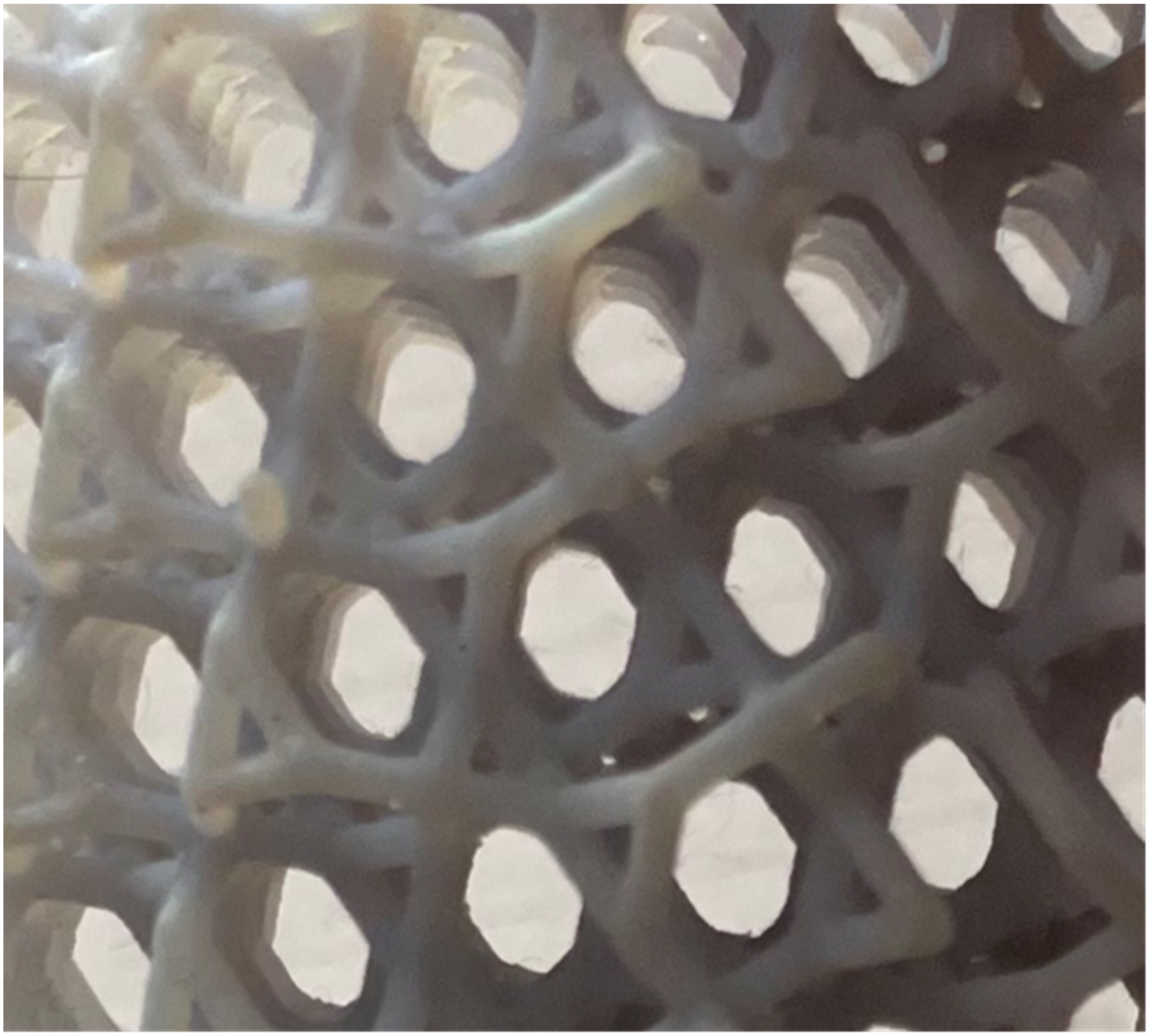

Fig. S15. **Visualization of flow channels through Laves structure.** Large scale, polymeric, printed Laves lattice, to highlight the channel structure in this ordered porous medium.

**Table S1**.

| **Temperature [°C]** | **D [$m^2s^{-1}$]** | $\boldsymbol{\eta_A/\eta_B}$ **[-]** |
|---|---|---|
| 35 | 3.3 x $10^{-11}$ | 7.2 |
| 55 | 4.9 x $10^{-11}$ | 5.8 |
| 75 | 8.5 x $10^{-11}$ | 2.3 |

Table S1: **Diffusion coefficient and relative viscosity of carbamate at various temperatures.** Carbamate diffusion coefficient and the ratio of viscosity of carbamate to amine at various temperatures

**Movie S1.**

**Flow of fluorescent beads in bulk and within the texture and roughness of the solid structure.** The flow of ~20-micrometer latex fluorescent beads demonstrates the existence of a thin layer of bulk liquid within the texture and roughness of the solid structure. See also Fig. 1

**Movie S2.**

**Experimental visualization of carbamate propagation in MEA during carbon capture.** Propagation of carbamate in MEA containing CCVJ at 35 °C. The bright zone is the high-viscosity area where carbamate molecules are present.

## REFERENCES AND NOTES

1. "IPCC, 2014: Climate Change 2014: Synthesis Report. Contribution of Working Groups I, II and III to the Fifth Assessment Report of the Intergovernmental Panel on Climate Change" (Intergovernmental Panel on Climate Change, 2014); https://www.ipcc.ch/site/assets/uploads/sites/2/2019/06/SR15_Full_Report_High_Res.pdf.

2. "Annual Energy Outlook 2011 with Projections to 2035", DOE/EIA-0383(2011), U.S. Energy Information Administration (EIA), 2011; https://doi.org/10.2172/1019039.

3. Y. Q. Fu, Y. B. Jiang, D. Dunphy, H. F. Xiong, E. Coker, S. Chou, H. X. Zhang, J. M. Vanegas, J. G. Croissant, J. L. Cecchi, S. B. Rempe, C. J. Brinker, Ultra-thin enzymatic liquid membrane for $CO_2$ separation and capture. *Nat. Commun.* **9**, 990 (2018).

4. T. Gelles, S. Lawson, A. A. Rownaghi, F. Rezaei, Recent advances in development of amine functionalized adsorbents for $CO_2$ capture. *Adsorption* **26**, 5–50 (2020).

5. T. M. McDonald, J. A. Mason, X. Q. Kong, E. D. Bloch, D. Gygi, A. Dani, V. Crocella, F. Giordanino, S. O. Odoh, W. S. Drisdell, B. Vlaisavljevich, A. L. Dzubak, R. Poloni, S. K. Schnell, N. Planas, K. Lee, T. Pascal, L. W. F. Wan, D. Prendergast, J. B. Neaton, B. Smit, J. B. Kortright, L. Gagliardi, S. Bordiga, J. A. Reimer, J. R. Long, Cooperative insertion of $CO_2$ in diamine-appended metal-organic frameworks. *Nature* **519**, 303–308 (2015).

6. J. J. Vericella, S. E. Baker, J. K. Stolaroff, E. B. Duoss, J. O. Hardin, J. Lewicki, E. Glogowski, W. C. Floyd, C. A. Valdez, W. L. Smith, J. H. Satcher, W. L. Bourcier, C. M. Spadaccini, J. A. Lewis, R. D. Aines, Encapsulated liquid sorbents for carbon dioxide capture. *Nat. Commun.* **6**, 6124 (2015).

7. K. Stephenne, Start-up of world's first commercial post-combustion coal fired CCS project: Contribution of Shell Cansolv to Saskpower boundary dam ICCS project, in *12th International Conference on Greenhouse Gas Control Technologies, Ghgt–12,* T. Dixon, H. Herzog, S. Twinning, Eds. (Energy Procedia, Elsevier Science Bv, 2014), vol. 63, pp. 6106–6110.

8. G. T. Rochelle, Amine scrubbing for $CO_2$ capture. *Science* **325**, 1652–1654 (2009).

9. J. R. Fair, A. F. Seibert, M. Behrens, P. P. Saraber, Z. Olujic, Structured packing performance-experimental evaluation of two predictive models. *Ind. Eng. Chem. Res.* **39**, 1788–1796 (2000).

10. N. Dave, T. Do, D. Palfreyman, P. H. M. Feron, Impact of liquid absorption process development on the costs of post-combustion capture in Australian coal-fired power stations. *Chem. Eng. Res. Des.* **89**, 1625–1638 (2011).

11. B. A. Oyenekan, G. T. Rochelle, Energy performance of stripper configurations for $CO_2$ capture by aqueous amines. *Ind. Eng. Chem. Res.* **45**, 2457–2464 (2006).

12. L. Dumee, C. Scholes, G. Stevens, S. Kentish, Purification of aqueous amine solvents used in post combustion $CO_2$ capture: A review. *Int. J. Greenh. Gas Control* **10**, 443–455 (2012).

13. F. Vega, F. M. Baena-Moreno, L. M. Gallego Fernández, E. Portillo, B. Navarrete, Z. Zhang, Current status of CO2 chemical absorption research applied to CCS: Towards full deployment at industrial scale. *Appl. Energy* **260**, 114313 (2020).

14. H. F. Bohn, W. Federle, Insect aquaplaning: Nepenthes pitcher plants capture prey with the peristome, a fully wettable water-lubricated anisotropic surface. *Proc. Natl. Acad. Sci. U.S.A.* **101**, 14138–14143 (2004).

15. S. Amini, S. Kolle, L. Petrone, O. Ahanotu, S. Sunny, C. N. Sutanto, S. Hoon, L. Cohen, J. C. Weaver, J. Aizenberg, N. Vogel, A. Miserez, Preventing mussel adhesion using lubricant-infused materials. *Science* **357**, 668–673 (2017).

16. H.-L. Girard, P. Bourrianne, D. Chen, A. Jaishankar, J. L. Vreeland, R. E. Cohen, K. K. Varanasi, G. H. McKinley, Asphaltene adsorption on functionalized solids. *Langmuir* **36**, 3894–3902 (2020).

17. J. Lee, S. Shin, Y. H. Jiang, C. Jeong, H. A. Stone, C. H. Choi, Oil-impregnated nanoporous oxide layer for corrosion protection with self-healing. *Adv. Funct. Mater.* **27**, 1606040 (2017).

18. P. Kim, T. S. Wong, J. Alvarenga, M. J. Kreder, W. E. Adorno-Martinez, J. Aizenberg, Liquid-infused nanostructured surfaces with extreme anti-ice and anti-frost performance. *ACS Nano* **6**, 6569–6577 (2012).

19. B. R. Solomon, K. S. Khalil, K. K. Varanasi, Drag reduction using lubricant-impregnated surfaces in viscous laminar flow. *Langmuir* **30**, 10970–10976 (2014).

20. X. Xu, C. Song, J. M. Andresen, B. G. Miller, A. W. Scaroni, Novel polyethylenimine-modified mesoporous molecular sieve of MCM-41 type as high-capacity adsorbent for $CO_2$ capture. *Energy Fuel* **16**, 1463–1469 (2002).

21. Y. Liu, Q. Ye, M. Shen, J. Shi, J. Chen, H. Pan, Y. Shi, Carbon dioxide capture by functionalized solid amine sorbents with simulated flue gas conditions. *Environ. Sci. Technol.* **45**, 5710–5716 (2011).

22. X. Feng, G. Hu, X. Hu, G. Xie, Y. Xie, J. Lu, M. Luo, Tetraethylenepentamine-Modified Siliceous Mesocellular Foam (MCF) for $CO_2$ Capture. *Ind. Eng. Chem. Res.* **52**, 4221–4228 (2013).

23. M. Yao, Y. Dong, X. Feng, X. Hu, A. Jia, G. Xie, G. Hu, J. Lu, M. Luo, M. Fan, The effect of post-processing conditions on aminosilane functionalizaiton of mesocellular silica foam for post-combustion CO2 capture. *Fuel* **123**, 66–72 (2014).

24. M. W. Hahn, M. Steib, A. Jentys, J. A. Lercher, Mechanism and kinetics of $CO_2$ adsorption on surface bonded amines. *J. Phys. Chem. C* **119**, 4126–4135 (2015).

25. G. Zhang, P. Zhao, Y. Xu, Z. Yang, H. Cheng, Y. Zhang, Structure Property–$CO_2$ capture performance relations of amine-functionalized porous silica composite adsorbents. *ACS Appl. Mater. Interfaces* **10**, 34340–34354 (2018).

26. J. D. Smith, R. Dhiman, S. Anand, E. Reza-Garduno, R. E. Cohen, G. H. McKinley, K. K. Varanasi, Droplet mobility on lubricant-impregnated surfaces. *Soft Matter* **9**, 1772–1780 (2013).

27. R. R. J. Sélo, S. Catchpole-Smith, I. Maskery, I. Ashcroft, C. Tuck, On the thermal conductivity of AlSi10Mg and lattice structures made by laser powder bed fusion. *Addit. Manuf.* **34**, 101214 (2020).

28. C. P. Mehnert, R. A. Cook, N. C. Dispenziere, M. Afeworki, Supported ionic liquid catalysis−A new concept for homogeneous hydroformylation catalysis. *J. Am. Chem. Soc.* **124**, 12932–12933 (2002).

29. J. P. Arhancet, M. E. Davis, J. S. Merola, B. E. Hanson, Hydroformylation by supported aqueous-phase catalysis: A new class of heterogeneous catalysts. *Nature* **339**, 454–455 (1989).

30. K. Sumida, D. L. Rogow, J. A. Mason, T. M. McDonald, E. D. Bloch, Z. R. Herm, T. H. Bae, J. R. Long, Carbon dioxide capture in metal-organic frameworks. *Chem. Rev.* **112**, 724–781 (2012).

31. A. Holewinski, M. A. Sakwa-Novak, C. W. Jones, Linking $CO_2$ sorption performance to polymer morphology in aminopolymer/silica composites through neutron scattering. *J. Am. Chem. Soc.* **137**, 11749–11759 (2015).

32. R. A. Davis, O. C. Sandall, CO2 CH4 separation by facilitated transport in amine-polyethylene glycol mixtures. *Aiche J.* **39**, 1135–1145 (1993).

33. R. G. Santiago, R. M. Siqueira, C. A. Alves, E. Vilarrasa-García, D. A. S. Maia, M. Bastos-Neto, D. C. S. de Azevedo, Evaluation of the thermal regeneration of an amine-grafted mesoporous silica used for CO2/N2 separation. *Adsorption* **26**, 203–215 (2020).

34. R. Hughes, G. Kotamreddy, A. Ostace, D. Bhattacharyya, R. L. Siegelman, S. T. Parker, S. A. Didas, J. R. Long, B. Omell, M. Matuszewski, Isotherm, kinetic, process modeling, and techno-economic analysis of a diamine-appended metal–organic framework for $CO_2$ Capture using fixed bed contactors. *Energy Fuel* **35**, 6040–6055 (2021).

35. J. Liu, P. K. Thallapally, B. P. McGrail, D. R. Brown, J. Liu, Progress in adsorption-based CO2 capture by metal–organic frameworks. *Chem. Soc. Rev.* **41**, 2308–2322 (2012).

36. Y. Belmabkhout, A. Sayari, Isothermal versus non-isothermal adsorption−desorption cycling of triamine-grafted pore-expanded MCM-41 Mesoporous Silica for $CO_2$ capture from Flue Gas. *Energy Fuel* **24**, 5273–5280 (2010).

37. A. Sayari, Y. Belmabkhout, Stabilization of amine-containing $CO_2$ adsorbents: Dramatic effect of water vapor. *J. Am. Chem. Soc.* **132**, 6312–6314 (2010).

38. E. J. Kim, R. L. Siegelman, H. Z. H. Jiang, A. C. Forse, J.-H. Lee, J. D. Martell, P. J. Milner, J. M. Falkowski, J. B. Neaton, J. A. Reimer, S. C. Weston, J. R. Long, Cooperative carbon capture and

steam regeneration with tetraamine-appended metal–organic frameworks. *Science* **369**, 392–396 (2020).

39. J. S. Wexler, I. Jacobi, H. A. Stone, Shear-driven failure of liquid-infused surfaces. *Phys. Rev. Lett.* **114**, 168301 (2015).

40. E. Miramontes, L. J. Love, C. Lai, X. Sun, C. Tsouris, Additively manufactured packed bed device for process intensification of CO2 absorption and other chemical processes. *Chem. Eng. J.* **388**, 124092 (2020).

41. R. B. Nielsen, M. D. Irvine, J. Mccullough, Controlling Corrosion In Amine Treating Plants, (1997)

42. R. E. Smallman, R. J. Bishop, Metals and Materials, in *Metals and Materials,* R. E. Smallman, R. J. Bishop, Eds. (Butterworth-Heinemann, 1995).

43. J. Bico, C. Tordeux, D. Quere, Rough wetting. *Europhys. Lett.* **55**, 214–220 (2001).

44. MAPS 4.3 Platform [Online], Scienomics SAS, 2020; http://www.scienomics.com.

45. W. D. Cornell, P. Cieplak, C. I. Bayly, I. R. Gould, K. M. Merz, D. M. Ferguson, D. C. Spellmeyer, T. Fox, J. W. Caldwell, P. A. Kollman, A second generation force field for the simulation of proteins, nucleic acids, and organic molecules. *J. Am. Chem. Soc.* **117**, 5179–5197 (1995).

46. M. Valiev, E. J. Bylaska, N. Govind, K. Kowalski, T. P. Straatsma, H. J. J. Van Dam, D. Wang, J. Nieplocha, E. Apra, T. L. Windus, W. A. de Jong, NWChem: A comprehensive and scalable open-source solution for large scale molecular simulations. *Comput. Phys. Commun.* **181**, 1477–1489 (2010).

47. S. Plimpton, Fast parallel algorithms for short-range molecular dynamics. *J. Comput. Phys.* **117**, 1–19 (1995).

48. J. R. Gissinger, B. D. Jensen, K. E. Wise, Modeling chemical reactions in classical molecular dynamics simulations. *Polymer* **128**, 211–217 (2017).

49. J. R. Gissinger, B. D. Jensen, K. E. Wise, REACTER: A heuristic method for reactive molecular dynamics. *Macromolecules* **53**, 9953–9961 (2020).

50. N. A. Rashidi, S. Yusup, A. Borhan, L. H. Loong, Experimental and modelling studies of carbon dioxide adsorption by porous biomass derived activated carbon. *Clean Technol. Environ. Policy* **16**, 1353–1361 (2014).

51. R. Chatti, A. K. Bansiwal, J. A. Thote, V. Kumar, P. Jadhav, S. K. Lokhande, R. B. Biniwale, N. K. Labhsetwar, S. S. Rayalu, Amine loaded zeolites for carbon dioxide capture: Amine loading and adsorption studies. *Microporous Mesoporous Mater.* **121**, 84–89 (2009).

52. A. Zhao, A. Samanta, P. Sarkar, R. Gupta, Carbon dioxide adsorption on amine-impregnated mesoporous SBA-15 Sorbents: Experimental and kinetics study. *Ind. Eng. Chem. Res.* **52**, 6480–6491 (2013).

53. M. Ojeda, M. Mazaj, S. Garcia, J. Xuan, M. M. Maroto-Valer, N. Z. Logar, Novel amine-impregnated mesostructured silica materials for $CO_2$ capture. *Energy Procedia* **114**, 2252–2258 (2017).

54. C. Zhou, S. Yu, K. Ma, B. Liang, S. Tang, C. Liu, H. Yue, Amine-functionalized mesoporous monolithic adsorbents for post-combustion carbon dioxide capture. *Chem. Eng. J.* **413**, 127675 (2021).

55. R. B. Bird, W. E. Stewart, E. N. Lightfoot, *Transport Phenomena* (John Wiley and Sons, Inc., 1961), pp. 780.

56. J. Sutharsan, D. Lichlyter, N. E. Wright, M. Dakanali, M. A. Haidekker, E. A. Theodorakis, Molecular rotors: Synthesis and evaluation as viscosity sensors. *Tetrahedron* **66**, 2582–2588 (2010).

57. G. Centeno, G. Sanchez-Reyna, J. Ancheyta, J. A. D. Munoz, N. Cardona, Testing various mixing rules for calculation of viscosity of petroleum blends. *Fuel* **90**, 3561–3570 (2011).

58. M. Teramoto, Approximate solution of facilitation factors for the transport of CO2 through a liquid membrane of amine solution. *Ind. Eng. Chem. Res.* **34**, 1267–1272 (1995).

59. S. H. Amirnordin, S. M. Seri, W. S.-I. W. Salim, H. A. Rahman, K. Hasnan, Pressure drop analysis of square and hexagonal cells and its effects on the performance of catalytic converters. *Int. J. Environ. Sci. Dev.* **2**, 239–247 (2011).
